# Production of Rainbow Colorants by Metabolically Engineered *Escherichia coli*

**DOI:** 10.1002/advs.202100743

**Published:** 2021-05-25

**Authors:** Dongsoo Yang, Seon Young Park, Sang Yup Lee

**Affiliations:** ^1^ Metabolic and Biomolecular Engineering National Research Laboratory, Systems Metabolic Engineering and Systems Healthcare Cross‐Generation Collaborative Laboratory, Department of Chemical and Biomolecular Engineering (BK21 plus program), Institute for the BioCentury Korea Advanced Institute of Science and Technology (KAIST) Daejeon 34141 Republic of Korea; ^2^ BioProcess Engineering Research Center KAIST Daejeon 34141 Republic of Korea; ^3^ BioInformatics Research Center KAIST Daejeon 34141 Republic of Korea

**Keywords:** membrane engineering, metabolic engineering, natural products, rainbow colorants, vesicle

## Abstract

There has been much interest in producing natural colorants to replace synthetic colorants of health concerns. *Escherichia coli* has been employed to produce natural colorants including carotenoids, indigo, anthocyanins, and violacein. However, production of natural green and navy colorants has not been reported. Many natural products are hydrophobic, which are accumulated inside or on the cell membrane. This causes cell growth limitation and consequently reduces production of target chemicals. Here, integrated membrane engineering strategies are reported for the enhanced production of rainbow colorants—three carotenoids and four violacein derivatives—as representative hydrophobic natural products in *E. coli*. By integration of systems metabolic engineering, cell morphology engineering, inner‐ and outer‐membrane vesicle formation, and fermentation optimization, production of rainbow colorants are significantly enhanced to 322 mg L^–1^ of astaxanthin (red), 343 mg L^–1^ of β‐carotene (orange), 218 mg L^–1^ of zeaxanthin (yellow), 1.42 g L^–1^ of proviolacein (green), 0.844 g L^–1^ of prodeoxyviolacein (blue), 6.19 g L^–1^ of violacein (navy), and 11.26 g L^–1^ of deoxyviolacein (purple). The membrane engineering strategies reported here are generally applicable to microbial production of a broader range of hydrophobic natural products, contributing to food, cosmetic, chemical, and pharmaceutical industries.

## Graphical abstract

Here, proviolacein, prodeoxyviolacein, violacein, and deoxyviolacein are produced in *Escherichia coli* as natural green, blue, navy, and purple colorants, in addition to astaxanthin, *β*‐carotene, and zeaxanthin known to be red, orange, and yellow colorants. Integration of systems metabolic engineering, cell morphology engineering, inner‐membrane, and outer‐membrane vesicle formation, and fermentation optimization for enhanced production of all seven rainbow colorants are described.

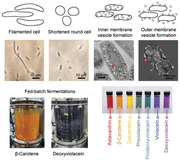

## 1 Introduction

Colorants have a profound impact on our lives as they have been widely employed in food additives, dyes, inks, and cosmetics, to name a few. Although many of them are directly related to human health either by oral intake (e.g., food additives) or transdermal absorption (e.g., cosmetics), most commercially produced synthetic colorants are chemically produced from petroleum. This could cause unexpected health problems, especially for children. To make food more attractive to children, vibrant synthetic colorants are extensively used (i.e., flavored snacks, drinks, candies),^[^ ^1^ ^]^ which is reported to be correlated with children's health risks such as hyperactive behaviors.^[^ ^2^ ^]^ Also, severe water pollution from fabric dyeing in textile industry is another problem; it was estimated that 17–20% of the entire industrial waste water is generated by dyeing or treating garments.^[^ ^3^ ^]^

To cope with the above‐mentioned concerns on well‐being and environmental concerns, systems metabolic engineering has emerged to allow development of microbial cell factories capable of sustainable and efficient production of value‐added chemicals and materials, including natural colorants.^[^ ^4^ ^]^ As many natural products contain chemical structures that can absorb different wavelengths of light (i.e., aromatic ring, conjugated system), they can display a wide array of colors, allowing them to be widely utilized as natural colorants since long before the emergence of petroleum‐based colorants. In contrast to the petroleum‐based synthetic colorants, natural colorants are environmentally friendly, involve less potential health‐related risks, and even possess beneficial pharmacological properties including anticancer, antibacterial, antiviral, anti‐inflammatory, and anti‐aging effects.^[^ ^5^ ^]^ These properties show that many natural colorants could be readily used as food colorants,^[^ ^6^ ^]^ active ingredients in cosmetics,^[^ ^7^ ^]^ or as general colorants including inks and textile dyes.^[^ ^8^ ^]^ Many of these natural colorants have been produced by metabolically engineered microorganisms, including betalains,^[^ ^9^ ^]^ anthocyanins,^[^ ^10^ ^]^ carotenoids,^[^ ^11^, ^12^ ^]^ and violacein derivatives.^[^ ^13^ ^]^ It has long been known that astaxanthin, *β*‐carotene, and zeaxanthin are red, orange, and yellow colorants. Indigo^[^ ^14^ ^]^ and indigoidine^[^ ^15^ ^]^ have been known to be blue colorants. Also, violacein derivatives have been reported to give purplish blue color.^[^ ^13^ ^]^ Some anthocyanin compounds give purple colors as well.^[^ ^10^ ^]^ However, natural compounds that can be used as green and navy colorants have not been reported before.

Natural colorants can be largely classified into hydrophilic and hydrophobic compounds which can be assessed by their characteristic logP values; higher logP of a compound indicates lower polarity of the compound. For example, betacyanin, a water‐soluble natural pigment has the logP value of −0.9, while *β*‐carotene which has a long hydrophobic carbon chain (C40) has the logP value of 13.5. The logP values can be predicted using the XLOGP3 software.^[^ ^16^ ^]^ When produced from microorganisms, hydrophilic compounds are diffused or secreted to the medium by transporters whereas hydrophobic compounds are accumulated inside the cell or within the cell membrane. In particular, high‐level production of hydrophobic compounds (i.e., carotenoids, bis‐indole pigments) has been problematic due to the limited innate capability of the cells to accumulate these compounds and their possible cytotoxic properties. Several recent studies attempted to resolve this issue by membrane engineering^[^ ^17^ ^]^ such as increasing the membrane area^[^ ^18^, ^19^ ^]^ or by forming intracellular lipid droplets which can serve as an intracellular reservoir for hydrophobic compounds.^[^ ^20^ ^]^ Although there have been preliminary reports on employing morphology engineering and formation of inner‐membrane vesicles (IMVs) or outer‐membrane vesicles (OMVs) for enhancing the production of hydrophobic compounds,^[^ ^18^, ^19^, ^20^, ^21^, ^22^, ^23^ ^]^ combinatorial applications of these membrane engineering strategies have not been reported. Despite these attempts, the titers of natural colorants need to be further increased for industrial applications.

**Figure 1:**
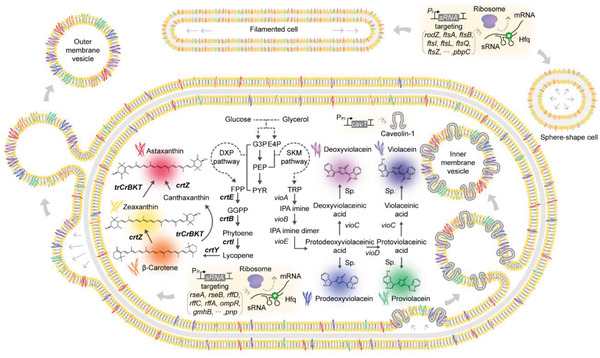
Overview of the metabolic engineering and membrane structure expansion strategies for the enhanced production of rainbow colorants (red, astaxanthin; orange, β‐carotene; yellow, zeaxanthin; green, proviolacein; blue, prodeoxyviolacein; navy, violacein; purple, deoxyviolacein). Morphology engineering was performed by knocking down the genes involved in cell division or cell wall metabolism. Inner‐membrane vesicles (IMVs) were formed by introducing the *cav1* gene encoding human caveolin‐1. Outer‐membrane vesicles (OMVs) were formed by knocking down the genes involved in OMV formation. The synthetic sRNA technology was employed to knockdown the expression levels of target genes by blocking translation. Bent arrow and T‐shape represent promoter and terminator, respectively. Solid and dotted lines represent single and multiple reactions, respectively. Abbreviations: G3P, glyceraldehyde 3‐phosphate; E4P, erythrose 4‐phosphate; PEP, phosphoenolpyruvate; PYR, pyruvate; DXP, 1‐deoxy‐D‐xylulose 5‐phosphate; SKM, shikimate; FPP, farnesyl diphosphate; GGPP, geranylgeranyl pyrophosphate; TRP, L‐tryptophan; IPA, indole pyruvate; Sp., spontaneous.

Here we report a strategy of integrating systems metabolic engineering, cell morphology engineering, IMV and OMV formation, and fermentation optimization for the production of seven natural colorants (including the first green colorant) covering the complete rainbow spectrum in *Escherichia coli* (**Figure** **1**). Carotenoids and violacein derivatives were chosen to cover all seven colors as they are important compounds in food, cosmetics, and drug industries due to many health‐related beneficial activities. Carotenoids belong to the category of terpenoids, which are the largest group of natural compounds produced from the C5 isoprene units. Carotenoids are responsible for the rich colors found in many plants (e.g., carrots and tomatoes) and algae, and are being actively used as anticancer, anti‐inflammatory, and antioxidant agents. Due to their beneficial health‐promoting properties and numerous applications, there has been an increasing number of studies on the production of carotenoids using microorganisms.^[^ ^24^ ^]^ Violacein derivatives are classified as bis‐indole pigments naturally produced from bacteria including *Chromobacterium violaceum* and *Janthinobacterium lividum*.^[^ ^25^ ^]^ These compounds display various pharmaceutical properties including antibacterial, antitumoral, antiviral, and antioxidant activities. They can also be used as sunscreen additives^[^ ^26^ ^]^ and other cosmetics applications, or as dyes for fabric.^[^ ^27^ ^]^ After the development of *E. coli* strains capable of efficiently producing rainbow colorants by metabolic engineering, morphology engineering, and vesicle engineering, fed‐batch fermentations were performed to evaluate production performances.

## 2 Results

### 2.1 Construction of Chassis Strains for the Production of Carotenoids by Colorimetric Screening

Among diverse carotenoid compounds, β‐carotene, zeaxanthin, and astaxanthin display orange, yellow, and red, respectively, the first three colors in the rainbow spectrum. Through the native 1‐deoxy‐D‐xylulose 5‐phosphate (DXP) pathway in *E. coli*, farnesyl diphosphate (FPP), a common precursor of carotenoids, can be produced. Introduction of *crtE* encoding geranylgeranyl pyrophosphate synthetase, *crtB* encoding phytoene synthase, and *crtI* encoding phytoene dehydrogenase (all from *Pantoea ananatis*) are required for lycopene production from FPP. Then, additional introduction of *crtY* encoding lycopene cyclase from *P. ananatis* leads to the production of β‐carotene. Subsequent introduction of *crtZ* encoding *β*‐carotene hydroxylase from *P. anantis* results in zeaxanthin production. For astaxanthin production, truncated *BKT* (*trCrBKT*) encoding β‐carotene ketolase from *Chlamydomonas reinhardtii* is additionally required (Figure 1).^[^ ^12^ ^]^

To optimize the metabolic flux toward target carotenoids and to minimize the accumulation of toxic intermediate lycopene, we constructed six libraries of 5′ untranslated region (5′UTR) designed by 5′UTR library designer software^[^ ^28^ ^]^ for each heterologous gene (*crtE*, *crtB*, *crtI*, *crtY*, *crtZ*, and *trCrBKT*), and examined their combinations to balance gene expression levels (see Text S1, Supporting Information, for details). The WLGB‐RPP strain, an *E. coli* K‐12 W3110 derivative that was previously constructed for lycopene overproduction,^[^ ^29^ ^]^ was used as a base strain. Since each carotenoid has distinct color, colorimetric screening was employed to easily screen overproducers. As a result of colorimetric screening, the strains BTC1, ZEA20, and ATX68 producing 18.7 mg L^–1^ of β‐carotene, 12.7 mg L^–1^ of zeaxanthin, and 14.5 mg L^–1^ of astaxanthin, respectively, in flask cultures were selected for further engineering. Glycerol, rather than glucose, was used as a carbon source as it led to higher production of carotenoids. This was thought to be due to the generation of more reducing equivalents which are required for the reactions in the carotenoids biosynthetic pathway,^[^ ^30^ ^]^ reduced aggregation of heterologous enzymes,^[^ ^31^ ^]^ or changes in the phospholipid composition of membrane that can contribute to the expansion of membrane space for the accumulation of carotenoids.^[^ ^32^ ^]^ As all three carotenoids share the same upstream pathway up to β‐carotene, BTC1 was selected as a representative strain for further engineering.

### 2.2 Construction of Chassis Strains for the Production of Violacein Derivatives

To complete the remaining rainbow color spectrum, metabolically engineered *E. coli* strains were developed to produce violacein derivatives. As the four violacein derivatives—prodeoxyviolacein, proviolacein, deoxyviolacein, and violacein—are reported to display distinct colors due to their different functional groups, engineered *E. coli* strains producing each of these compounds were constructed to compare their colors. Since all of the four violacein derivatives are produced from L‐tryptophan (Figure 1A), the IND5 strain harboring pTacGEL, which we previously developed for driving strong metabolic flux toward L‐tryptophan,^[^ ^14^ ^]^ was employed. Using this strain as a base strain, heterologous biosynthetic pathways from *C. violaceum* were introduced for the production of violacein derivatives.

First, the complete violacein biosynthetic gene cluster (BGC) *vioABCDE* was introduced into IND5 (pTacGEL) to construct the strain VIO, leading to the production of 453 mg L^–1^ violacein (together with 44.2 mg L^–1^ deoxyviolacein) from glucose (Figure S2B, Supporting Information). Regarding the carbon source, it was previously suggested that glucose might inhibit violacein production^[^ ^33^ ^]^ and glycerol was suggested as a better carbon source for deoxyviolacein production as the metabolic pathway from glycerol to glyceraldehyde 3‐phosphate, the direct precursor of the tryptophan pathway, is shorter than that from glucose.^[^ ^34^ ^]^ Thus, glycerol was also tested as a sole carbon source. Switching the carbon source from glucose to glycerol significantly increased violacein production (1.36 g L^–1^ violacein together with 0.130 g L^–1^ deoxyviolacein; *P* = 5.5 × 10^–5^) (Figure S2B, Supporting Information). Then, each of the *vioABCE*, *vioABDE*, and *vioABE* BGCs was introduced into IND5 (pTacGEL) to construct the strains DVIO, PVIO, and PDVIO, respectively. Each of these strains produced 1.04 g L^–1^ of deoxyviolacein, 151 mg L^–1^ of proviolacein, and 114 mg L^–1^ of prodeoxyviolacein from glycerol, respectively (Figure S2C,D, Supporting Information). The colors of the four violacein derivatives after extraction by dimethylsulfoxide (DMSO) are found to be as follows: proviolacein, green; prodeoxyviolacein, blue; violacein, navy; deoxyviolacein, purple (Figure 1B; Figure S2G, Supporting Information). To the best of our knowledge, this is the first report on the production of a natural green colorant. As all four violacein derivatives have similar chemical and physical properties and share L‐tryptophan as the common precursor, deoxyviolacein was selected as the representative strain to test further engineering strategies.

### 2.3 Altering Cell Morphology by Deregulation of Membrane‐Associated Metabolism

**Figure 2:**
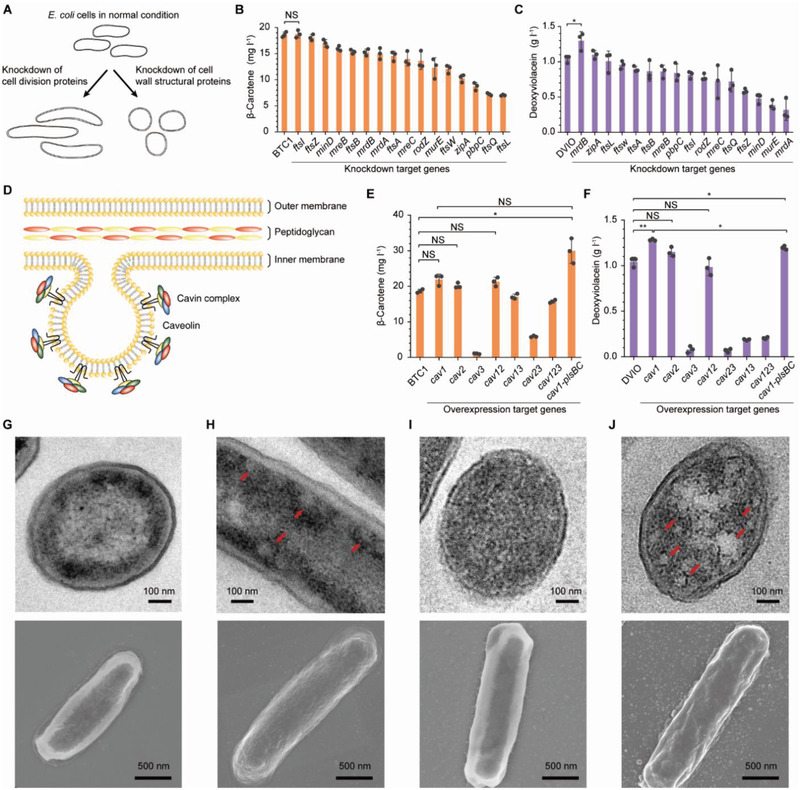
Morphology engineering and IMV formation for the enhanced production of rainbow colorants. A) Schematic representation of morphology engineering to expand the space in which rainbow colorants can be accumulated. Knockdown of genes involved in cell division would lead to elongated cells whereas knockdown of genes involved in cell wall synthesis or maintenance would lead to shorter cells with spherical and irregular shapes. The synthetic sRNA technology was employed to knockdown the expression levels of target genes by blocking translation. B) β‐Carotene production in the engineered strains introduced with the sRNAs targeting genes related to cellular morphology. C) Deoxyviolacein production in the engineered strains introduced with the sRNAs targeting genes related to cellular morphology. D) Schematic representation of employing IMVs (caveolae). IMVs were formed by introducing the *cav1* gene encoding human caveolin‐1. E) β‐Carotene production by employing IMVs. F) Deoxyviolacein production by employing IMVs. TEM (upper panels) and SEM (lower panels) images of G) the control β‐carotene producer BTC1, H) BTC1 expressing *cav1*, I) the control deoxyviolacein producer deoxyviolacein, and J) deoxyviolacein expressing *cav1* are shown. For panels (H and I), red arrows represent IMVs. Error bars are mean ± SD (standard deviation; *n* = 3). B,C) *P < 0.05, determined by two‐tailed Student's *t*‐test. E,F) *P < 0.01, **P < 0.002, determined by two‐tailed Student's *t*‐test. *P*‐value thresholds were adjusted using Bonferroni correction (corrected significance levels represented as *α*/*m*; *α*, original significance level; *m*, number of hypotheses). NS, not significant.

Carotenoids are hydrophobic as their chemical structures have a common C_40_ backbone. Violacein derivatives are also hydrophobic due to their hydrophobic carbon‐ring moieties. These hydrophobic natural products are either integrated into the cell membrane or accumulated on the cell membrane, rather than being secreted to the culture medium.^[^ ^35^, ^36^ ^]^ Thus, expanding membrane capacity was expected to enhance the accumulation of carotenoids and violacein derivatives, as demonstrated for zeaxanthin production.^[^ ^37^ ^]^ We sought to expand membrane capacity by deregulation of membrane‐associated metabolism which would alter cell morphology (**Figure** **2**A). As reported previously, repression of cell division‐related genes (i.e., *ftsZ*) would elongate the cells by blocking cell division.^[^ ^38^ ^]^ On the other hand, repression of genes related to cell wall synthesis or maintenance (i.e., *mreB*) would alter the cell morphology into different shapes (usually spherical) as cell wall is responsible for maintaining the rod shape of *E. coli*.^[^ ^38^ ^]^ It was thus expected that knocking down the genes belonging to these two categories would increase the total membrane area per cell.

Repression of target gene expression was achieved by employing the synthetic small regulatory RNA (sRNA) technology.^[^ ^39^, ^40^ ^]^ The synthetic sRNA is a trans‐acting target gene knockdown tool comprising a target‐specific small RNA and Hfq protein, and is particularly useful for the knockdown of multiple gene targets including essential genes to the desired levels.^[^ ^39^ ^]^ From the *E. coli* genome, nine knockdown target genes related to cell division regulation (*ftsABILQWZ*, *minD*, and *zipA*) and seven knockdown target genes related to cell wall synthesis or cell shape maintenance (*rodZ*, *mrdAB*, *mreBCE*, *murE*, and *pbpC*) were selected (Table S4, Supporting Information). The sRNAs corresponding to these target genes were obtained from the previously constructed *E. coli* genome‐scale sRNA library,^[^ ^41^ ^]^ which were transformed into the BTC1 and DVIO strains for subsequent flask cultivation. When the cell division‐related genes were repressed, elongated cells were observed for both strains (Figure S3, Supporting Information). However, none of them showed increased production of β‐carotene or deoxyviolacein (Figure 2B,C). When the genes related to cell wall synthesis or maintenance were repressed, shorter cells with spherical and irregular shapes were observed in both BTC1 and DVIO (Figure S3, Supporting Information). Knockdown of these target genes was not effective in enhancing β‐carotene production (Figure 2B). However, knockdown of *mrdB* (encoding a cell wall shape‐determining protein) in DVIO led to 25% increase in deoxyviolacein production (1.30 g L^–1^; *P* = 0.032; Figure 2C) when compared to that (1.04 g L^–1^) produced by the base strain. Increased formation of deoxyviolacein crystals inside and outside the cells could be identified through microscopy observation of the DVIO strain with *mrdB* knockdown (Figure S3B, Supporting Information).

### 2.4 Generation of IMVs for Colorants Accumulation

To further expand membrane structures, eukaryotic IMVs were employed. Of several eukaryotic vesicles reported, caveolae are small invaginations in the plasma membrane of animal cells, playing essential roles in many important cellular metabolisms including endocytosis and signal transduction (Figure 2D).^[^ ^42^ ^]^ Of several proteins and lipids that comprise caveolae, caveolin proteins (Cav1, Cav2, and Cav3) are important in the formation of globular structure. It has been previously reported that heterologous expression of *cav1* resulted in successful formation of caveolae in *E. coli* whereas introduction of *cav2* led to the formation of irregular membrane structures inside the cell.^[^ ^43^ ^]^ Heterologous expression of *cav3* has not yet been reported. Cav1 has also been employed to increase the accumulation of membrane proteins in *E. coli* ^[^ ^21^ ^]^ or to enhance the uptake and conversion of fatty acids into esters.^[^ ^22^ ^]^ We thus examined whether the production of rainbow colorants can be enhanced by expanding membrane structures by generation of IMVs through employing caveolae.

First, three genes encoding different caveolin proteins—*cav1*, *cav2*, and *cav3*—from *Homo sapiens* were individually cloned into pTrc99A (see Text S2, Supporting Information, for the sequences of the codon‐optimized genes). Each of the three plasmids was transformed into the BTC1 and DVIO strains to examine their effects on colorants production. Cav1 was the most effective caveolin protein in enhancing the production of both colorants. Upon expression of *cav1*, β‐carotene production was increased to 21.9 mg L^–1^ (*P* = 0.027) and deoxyviolacein to 1.28 g L^–1^ (*P* = 1.5 × 10^–3^) (Figure 2E,F). Expression of *cav2* also slightly enhanced β‐carotene production to 20.2 mg L^–1^ (*P* = 0.045), but deoxyviolacein production (1.15 g L^–1^) was not affected (*P* = 0.062). Expression of *cav3* rather decreased both the β‐carotene titer (1.04 mg L^–1^; *P* = 4.8 × 10^–7^) and the deoxyviolacein titer (79.0 mg L^–1^; *P* = 1.3 × 10^–5^; Figure 2E,F) significantly. Simultaneous expression of the *cav1* and *cav2* genes resulted in increased β‐carotene production (21.3 mg L^–1^; *P* = 0.029) compared to the base strain, while deoxyviolacein production (0.980 g L^–1^) was not affected (*P* = 0.42). The combinatorial expression of *cav23*, *cav13*, and *cav123* resulted in decreased titers of both colorants when compared with those obtained from the base strains (Figure 2E,F).

Transmission electron microscopy (TEM) analysis was performed to observe the intracellular structures (caveolae) formed by the expression of *cav1* (Figure 2G–J). Scanning electron microscopy (SEM) analysis was also performed to examine whether any changes in outer membrane occurred. It was confirmed that only intracellular membrane structures were formed upon *cav1* expression (Figure 2G–J). Next, it was pursued to increase membrane lipid supply by overexpressing the *E. coli* *plsBC* genes, which are responsible for the conversion of acyl‐ACP and glycerol 3‐phosphate into diacylglycerol 3‐phosphate.^[^ ^19^ ^]^ As expected, β‐carotene production was further increased to 29.9 mg L^–1^ (*P* = 0.022), which was higher than that obtained with the strain only harboring the *cav1* gene. In the case of deoxyviolacein production, overexpression of *plsBC* together with *cav1* resulted in a deoxyviolacein titer (1.20 g L^–1^; *P* = 8.9 × 10^–3^) higher than that (1.04 g L^–1^) obtained with the DVIO strain, but lower than that obtained by *cav1* overexpression only (1.28 g L^–1^; *P* = 3.1 × 10^–3^) (Figure 2E,F). Whereas carotenoids such as β‐carotene are known to be incorporated into the cell membrane,^[^ ^44^ ^]^ bis‐indole pigments such as deoxyviolacein are known to form crystals that are accumulated around the cell membrane.^[^ ^35^ ^]^ Thus, increased phospholipid supply by overexpression of *plsBC* would have directly affected the production of β‐carotene whereas its effect on the production of deoxyviolacein would have been marginal.

### 2.5 Generation of OMVs for Colorants Production

**Figure 3:**
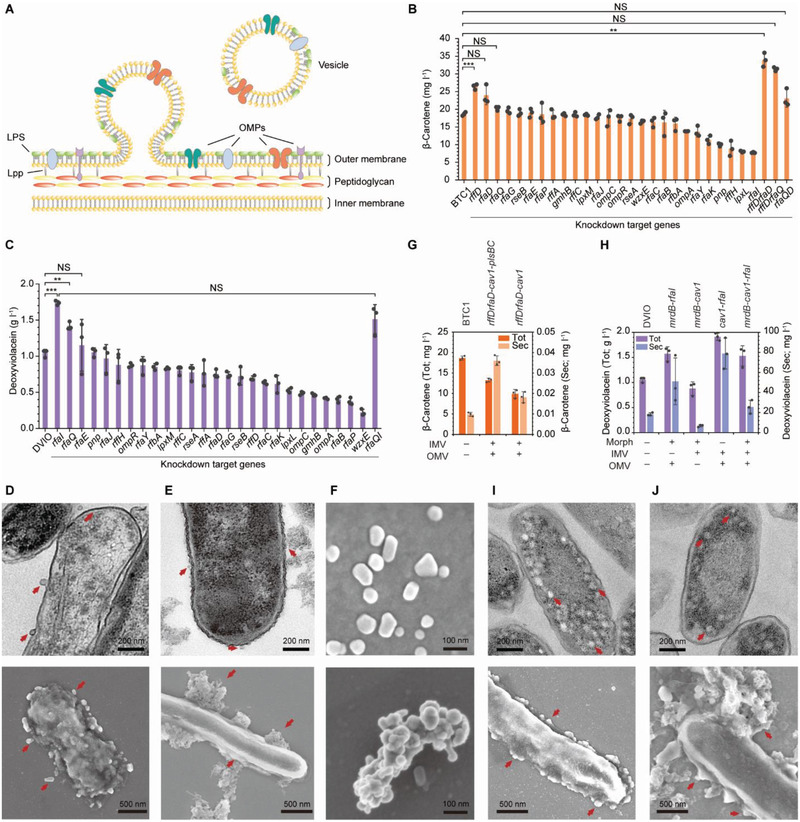
Formation of OMVs for the enhanced production of rainbow colorants. A) Schematic representation of OMVs formation. OMVs were formed by repression of genes encoding outer‐membrane proteins, repression of genes related to outer‐membrane or peptidoglycan integrity, or activation of the σ^E^ factor. The synthetic sRNA technology was employed to knockdown the expression levels of target genes by blocking translation. Abbreviations are OMP, outer‐membrane protein; LPS, lipopolysaccharide. B) β‐Carotene production by employing OMVs. *P < 0.0083, **P < 0.0017, ***P < 0.00017, determined by two‐tailed Student's *t*‐test. C) Deoxyviolacein production by employing IMVs. *P < 0.0125, **P < 0.0025, ***P < 0.00025, determined by two‐tailed Student's *t*‐test. TEM (upper panels) and SEM (lower panels) images of D) BTC1 introduced with anti‐*rffD* sRNA and E) DVIO introduced with anti‐*rfaI* sRNA. F) SEM images of purified OMVS from BTC1 harboring anti‐*rffD* sRNA (upper panel) and DVIO harboring anti‐*rfaI* sRNA (lower panel). TEM (upper panels) and SEM (lower panels) images of I) BTC1 introduced with anti‐*rffD* and anti‐*rfaD* sRNAs and *cav1*‐*plsBC* and J) DVIO introduced with anti‐*rfaI* sRNA and *cav1*. G) β‐Carotene production by testing synergistic effects of employing the strategies of forming IMVs and OMVs. H) Deoxyviolacein production by testing synergistic effects of employing the strategies of morphology engineering, IMVs formation, and OMVs formation. Abbreviations in (G,H) are Tot, total titer; Sec, the titer obtained from the extracellular medium; Morph, morphology engineering. For panels (D–J), Red arrows represent IMVs or OMVs. Error bars are mean ± SD (*n* = 3). *P*‐value thresholds were adjusted using Bonferroni correction (*P* < *α*/*m*). NS, not significant.

Due to the limited membrane capacity for the hydrophobic natural products, their maximum titers cannot reach beyond the maximum storage space inside the cell. Moreover, excess intracellular accumulation of these natural products can cause metabolic burden to the host cells and negatively affect cellular physiology and metabolism, which would consequently lead to the inhibition of both cell growth and product formation. For this reason, extracellular vesicles were also employed to further expand the membrane structures and to secrete the accumulated colorants to the extracellular medium. In gram‐negative bacteria, OMVs are generated for transportation of genetic materials, cell detoxification, and bacterial communication, among many other purposes.^[^ ^45^ ^]^ In contrast to IMVs, genes that can explicitly generate OMVs in bacteria have not been identified. It has been reported that repression of genes encoding outer‐membrane proteins, repression of genes related to outer‐membrane or peptidoglycan integrity, or activation of the σ^E^ factor can induce OMV formation (**Figure** **3**A).^[^ ^23^, ^46^ ^]^ Thus, we sought to generate OMVs in *E. coli* using the synthetic sRNA technology in order to test all the three hypotheses.^[^ ^39^ ^]^

The selected 26 knockdown target genes are as follows (Table S4, Supporting Information): genes encoding outer‐membrane proteins (*ompA*, *ompC*, and *ompR*), genes involved in outer‐membrane/peptidoglycan integrity (*rffA*, *rffC*, *rffD*, *gmhB*, *lpxL*, *lpxM*, *rfaB*, *rfaC*, *rfaD*, *rfaE*, *rfaG*, *rfaI*, *rfaJ*, *rfaK*, *rfaP*, *rfaQ*, *rfaY*, *rfbA*, *rffH*, *wzxE*, and *pnp*), and genes encoding anti‐σ^E^ factors (*rseA* and *rseB*). From the *E. coli* synthetic sRNA library,^[^ ^40^ ^]^ the 26 selected sRNAs were introduced into the BTC1 and the DVIO strains and were cultured in flasks (Figure 3B,C). When measuring the concentrations of β‐carotene and deoxyviolaein, those in both cell pellets and extracellular medium were measured. In the BTC1 strain, sRNA‐based knockdown of *rffD* encoding UDP‐N‐acetyl‐D‐mannosamine dehydrogenase, *rfaD* encoding ADP‐L‐glycero‐D‐manno‐heptose‐6‐epimerase, and *rfaQ* encoding lipopolysaccharide (LPS) core biosynthesis protein (heptosyltransferase III) was effective in enhancing the β‐carotene production to 26.4 mg L^–1^ (*P* = 1.4 × 10^–4^), 24.2 mg L^–1^ (*P* = 0.024), and 20.3 mg L^–1^ (*P* = 0.022), respectively (Figure 3B). These correspond to 41.6%, 29.7%, and 8.74% increase of the β‐carotene titer, respectively, from that (18.7 mg L^–1^) obtained with the BTC1 strain. The *rffD* gene is involved in the biosynthesis of enterobacterial common antigen (one of outer‐membrane components) and *rfaD* and *rfaQ* are both involved in the biosynthesis of LPS, which are important for the stability and the architecture of the outer‐membrane. Thus, downregulation of these genes would have disrupted the integrity of cell outer‐membrane, and consequently promoted OMV formation providing enhanced β‐carotene accumulation capacity. Next, in order to further increase OMV formation and thus β‐carotene production, different combinations of anti‐*rffD*, ‐*rfaD*, and ‐*rfaQ* sRNA cassettes were introduced to the BTC1 strain and cultured in flasks. As a result, BTC1 (pWAS‐anti‐*rffDrfaD*) and BTC1 (pWAS‐anti‐*rffDrfaQ*) produced higher concentrations of β‐carotene (34.2 and 31.5 mg L^–1^, respectively) compared with the strains having single gene repression (Figure 3B); the highest β‐carotene titer obtained with the BTC1 (pWAS‐anti‐*rffDrfaD*) strain corresponds to 83.3% increase of the titer (*P* = 1.9 × 10^–4^) compared to that (18.7 mg L^–1^) obtained with the BTC1 strain.

In the DVIO strain, sRNA‐based knockdown of *rfaQ* and *rfaI* encoding UDP‐D‐galactose:(glucosyl)lipopolysaccharide‐alpha‐1,3‐D‐galactosyltransferase enhanced deoxyviolacein production to 1.42 g L^–1^ (*P* = 0.001) and 1.74 g L^–1^ (*P* = 3.9 × 10^–5^), respectively (Figure 3C). These correspond to 36.5% and 67.3% increase of the deoxyviolacein titer, respectively, when compared with that (1.04 g L^–1^) obtained with the base DVIO strain. Downregulation of the LPS biosynthesis was effective in enhancing deoxyviolacein production, as in β‐carotene production. To test if the two selected knockdown target genes can synergistically enhance deoxyviolacein production, an sRNA expression vector harboring both anti‐*rfaQ* and anti‐*rfaI* sRNA cassettes (pWAS‐anti‐*rfaQrfaI*) was introduced into DVIO and was cultured in baffled flasks. Although deoxyviolacein titer (1.51 g L^–1^) increased compared with that of the control DVIO strain (1.04 g L^–1^; *P* = 0.017), it was lower than that (1.74 g L^–1^) obtained with the *rfaI*‐knockdown strain (Figure 3C).

Next, similarly to IMV formation studies above, the *plsBC* genes were overexpressed in addition to OMV formation to see whether increasing the diacylglycerol 3‐phosphate pool would further enhance colorants production.^[^ ^19^ ^]^ When pTrc99A‐*plsBC* was introduced into BTC1 (pWAS‐anti‐*rffD*), BTC1 (pWAS‐anti‐*rfaD*), BTC1 (pWAS‐anti‐*rfaQ*), and DVIO (pWAS‐anti‐*rfaI*), however, both β‐carotene and deoxyviolacein titers were significantly reduced (Figure S4F,G, Supporting Information).

To assess the portions of β‐carotene and deoxyviolacein secreted to the medium, cell‐free media obtained from the above cultures were extracted and analyzed by high‐performance liquid chromatography (HPLC). The OMVs from the cell‐free media were also separately extracted and analyzed. Unexpectedly, the concentrations of β‐carotene secreted to the medium were much less than the observed increase of the total β‐carotene concentrations (Figure 3B; Figure S4A, Supporting Information). In the case of the BTC1 (pWAS‐anti‐*rffDrfaD*) strain, the best β‐carotene producer, the portion of β‐carotene secreted was only 0.72% (Figure S4A, Supporting Information) although β‐carotene secreted (0.25 mg L^–1^) was significantly higher (*P* = 2.7 × 10^–4^) than that (0.01 mg L^–1^) from the BTC1 strain. The β‐carotene produced and excreted through OMVs was 0.160 mg L^–1^ (Figure S4B, Supporting Information). Similarly, only a little portion of deoxyviolacein was found in the medium (75.3 mg L^–1^, 4.3% of the total deoxyviolacein produced; Figure S4C, Supporting Information) or in the OMVs (40.2 mg L^–1^; Figure S4D, Supporting Information). After observing the DVIO (pWAS‐anti‐*rfaI*) strain by optical microscopy, it was hypothesized that such low deoxyviolacein secretion was not due to the inefficient formation of OMVs, but rather due to the aggregation of the OMVs and concomitant formation of extracellular deoxyviolacein crystals (Figure S4E, Supporting Information).

To confirm the hypothesis and to more precisely observe the intracellular and extracellular membrane structures of BTC1 (pWAS‐anti‐*rffDrfaD*) and DVIO (pWAS‐anti‐*rfaI*), TEM and SEM analyses were performed. Formation of OMVs and highly wrinkled outer‐membrane morphology were observed for both BTC1 (pWAS‐anti‐*rffDrfaD*) (Figure 3D, upper panel) and DVIO (pWAS‐anti‐*rfaI*) (Figure 3E, upper panel) by TEM, showing clear evidences of vascularization. Formation of OMVs from BTC1 (pWAS‐anti‐*rffDrfaD*) (Figure 3D, lower panel) and DVIO (pWAS‐anti‐*rfaI*) (Figure 3E, lower panel) was also observed by SEM. Also, significant amount of OMVs and their aggregates that were not completely separated from the cell surface were observed by TEM and SEM, explaining the reason for low β‐carotene and deoxyviolacein concentrations in the extracellular medium (Figure 3D,E). The purified OMVs were also examined by SEM. For the BTC1 (pWAS‐anti‐*rffDrfaD*) strain, formation of small OMVs (the sizes of ≈50–60 nm) was observed (Figure 3F, upper panel). On the other hand, for the DVIO (pWAS‐anti‐*rfaI*) strain, the OMV aggregates (that were also observed from optical microscopy; Figure S4E, Supporting Information) were observed, confirming that OMV formation resulted in abundant formation of vesicle aggregates and deoxyviolacein crystals outside the cell (Figure 3F, lower panel).

### 2.6 Integration of Membrane Expansion Strategies for Enhanced Production of Rainbow Colorants

Having observed beneficial effects of morphology engineering, IMV formation, and OMV formation, we next examined whether combination of these strategies can further enhance the production of rainbow colorants. The knockdown and overexpression target genes that enabled the highest increase in colorants production were selected to assess their combinatorial effects on β‐carotene and deoxyviolacein production using the representative producer strains BTC1 and DVIO. In the case of the BTC1 strain, the best OMV‐forming strategy (knockdown of *rffD* and *rfaD*) and the best IMV‐forming strategy (overexpression of *cav1* and *plsBC*) were tested in combinations since membrane morphology engineering was not beneficial. As overexpression of *plsBC* enhanced β‐carotene production in the IMV‐forming strain whereas reduced β‐carotene production in the OMV‐forming strain, the combination without *plsBC* overexpression was also tested (Figure 3G). In contrast to what was expected, simultaneous formation of IMVs and OMVs resulted in decreased β‐carotene production even compared with the base BTC1 strain. In the case of the DVIO strain, the best morphology engineering strategy (knockdown of *mrdB*), the best IMV‐forming strategy (overexpression of *cav1*), and the best OMV‐forming strategy (knockdown of *rfaI*) were tested in combinations. Differently from the β‐carotene production case, the highest deoxyviolacein titer (1.89 g L^–1^) was obtained by simultaneous formation of IMVs and OMVs, which corresponds to 81.7% increase of the titer (*P* = 8.0 × 10^–5^) compared with that (1.04 g L^–1^) obtained with the DVIO strain (Figure 3H). TEM and SEM analyses were performed to examine the intracellular and extracellular membrane structures of BTC1 (pWAS‐anti‐*rffDrfaD*‐*cav1*‐*plsBC*) and DVIO (pWAS‐anti‐*rfaI*‐*cav1*) strains. The simultaneous formation of IMVs and OMVs was observed in both strains (Figure 3I,J).

After developing membrane engineering strategies for the enhanced production of β‐carotene and deoxyviolacein, the most effective strategies were applied to the production of the other five colorants. Since the IMV‐forming strategy (overexpression of *cav1* and *plsBC*) and the OMV‐forming strategy (knockdown of *rffD* and *rfaD*) were all highly effective in enhancing β‐carotene production, these strategies were applied to ZEA20 (the base zeaxanthin producing strain) and ATX68 (the base astaxanthin producing strain). The strategy of OMV formation resulted in higher titers of zeaxanthin (18.4 mg L^–1^; *P* = 1.2 × 10^–5^) and astaxanthin (22.7 mg L^–1^; *P* = 2.2 × 10^–5^) (Figure S4H,I, Supporting Information). In the case of deoxyviolacein, the combination of the IMV‐forming strategy (overexpression of *cav1*) and the OMV‐forming strategy (knockdown of *rfaI*) was the most effective. Since employing the IMV‐forming strategy and the OMV‐forming strategy individually were also highly effective, all three strategies were applied to PDVIO (the base prodeoxyviolacein producing strain), PVIO (the base proviolacein strain), and VIO (the base violacein producing strain) (Figure S4J‐L, Supporting Information). The combined strategy of IMV and OMV formation by the overexpression of *cav1* and knockdown of *rfaI* resulted in the highest titers of proviolacein (402 mg L^–1^; *P* = 2.6 × 10^–4^) and violacein (2.84 g L^–1^; *P* = 1.1 × 10^–3^) (Figure S4K,L, Supporting Information). In the case of prodeoxyviolacein, the OMV‐forming strategy resulted in the highest titer of 341 mg L^–1^ (*P* = 2.0 × 10^–3^), while the combined IMV‐OMV‐forming strategy also increased the titer to 301 mg L^–1^ (*P* = 1.4 × 10^–3^), significantly higher than that (114 mg L^–1^) obtained with the control PDVIO strain (Figure S4J, Supporting Information).

**Figure 4:**
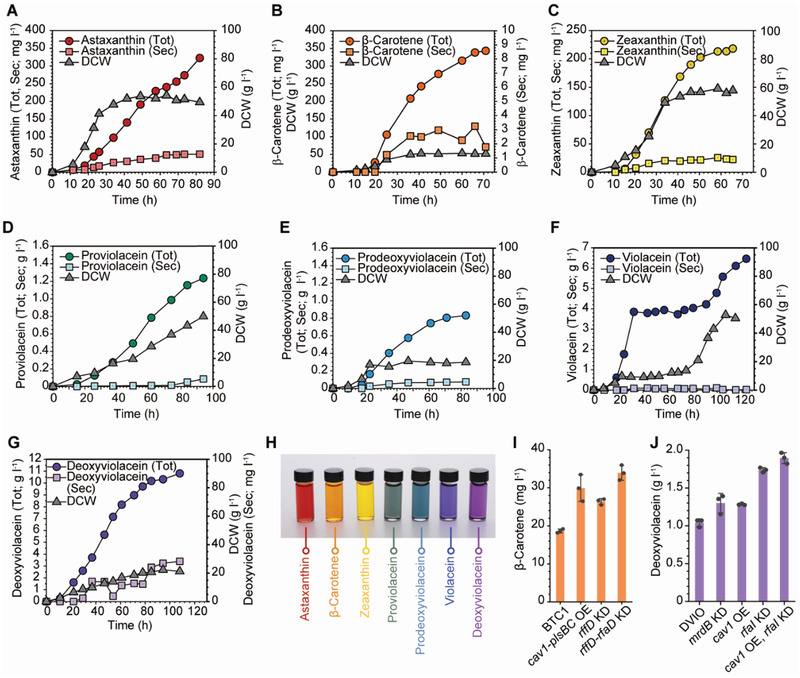
Time profiles of the fed‐batch fermentation of engineered strains producing seven rainbow colorants. Fed‐batch fermentation profiles of A) ATX68 (pWAS‐anti‐*rffDrfaD*) producing astaxanthin, B) BTC1 (pWAS‐anti‐*rffDrfaD*) producing β‐carotene, C) ZEA20 (pWAS‐anti‐*rffDrfaD*) producing zeaxanthin, D) PVIO (pWAS‐anti‐*rfaI*‐*cav1*) producing proviolacein, E) PDVIO (pWAS‐anti‐*rfaI*) producing prodeoxyviolacein, F) VIO (pWAS‐anti‐*rfaI*‐*cav1*) producing violacein, and G) DVIO (pWAS‐anti‐*rfaI*‐*cav1*) producing deoxyviolacein are shown. (H) Picture of rainbow colorants produced by the engineered strains. Each colorant was appropriately diluted in chloroform (β‐carotene), acetone (zeaxanthin), or DMSO (astaxanthin and violacein derivatives). The concentrations of the colorants shown in the picture are: red, 43.3 mg L^–1^ astaxanthin; orange, 80.5 mg L^–1^ *β*‐carotene; yellow, 14.9 mg L^–1^ zeaxanthin; green, 6.56 mg L^–1^ proviolacein; blue, 5.51 mg L^–1^ prodeoxyviolacein; navy, mixture of 59.7 mg L^–1^ violacein and 3.8 mg L^–1^ deoxyviolacein; purple, 46.9 mg L^–1^ deoxyviolacein. Summary of the titers of I) β‐carotene and J) deoxyviolacein obtained by flask culture of the strains applied with the major strategies in this study. Abbreviations are Tot, total titer; Sec, the titer obtained from extracellular medium; DCW, dry cell weight; KD, knockdown; OE, overexpression.

Finally, fed‐batch cultures were performed in a 6.6 L bioreactor to examine the performances of the seven constructed strains producing rainbow colorants. For each color product, the engineered strain showing the highest titer in flask culture was employed. After optimization of fermentation conditions (Text S3, Supporting Information), the following titers of carotenoids and violacein derivatives were obtained: 322 mg L^–1^ of astaxanthin in 82 h using ATX68 (pWAS‐anti‐*rffDrfaD*); 343 mg L^–1^ of β‐carotene in 71 h using BTC1 (pWAS‐anti‐*rffDrfaD*); 218 g L^–1^ of zeaxanthin in 65.5 h using ZEA20 (pWAS‐anti‐*rffDrfaD*); 1.30 g L^–1^ of proviolacein in 94 h using PVIO (pWAS‐anti‐*rfaI*‐*cav1*); 0.855 g L^–1^ of prodeoxyviolacein in 83 h using PDVIO (pWAS‐anti‐*rfaI*); 6.69 g L^–1^ of violacein (together with 1.39 g L^–1^ of deoxyviolacein) in 123.5 h using VIO (pWAS‐anti‐*rfaI*‐*cav1*); 11.3 g L^–1^ of deoxyviolacein in 108 h using DVIO (pWAS‐anti‐*rfaI*‐*cav1*) (**Figure** **4**; for detailed methods, see the Experimental Section). The time‐lapse movies of the whole fermentation processes of BTC1 (pWAS‐anti‐*rfaDrffD*) and VIO (pWAS‐anti‐*rfaI*‐*cav1*) are presented as Movies S1 and S2, Supporting Information, respectively, for the readers. To examine the reproducibility, all fed‐batch fermentations were repeated independently once more; the results were reproducible (Figure S5, Supporting Information). The titer, productivity, and content of the rainbow colorants produced by seven different strains are summarized in Table S5, Supporting Information. The rainbow colorants produced were purified and pictured together (Figure 4H).

## 3 Conclusion

In this study, we developed metabolically engineered *E. coli* strains capable of producing seven rainbow colorants. Proviolacein, prodeoxyviolacein, violacein, and deoxyviolacein were produced as natural green, blue, navy, and purple colorants, respectively, in addition to the well‐studied colorants, astaxanthin (red), β‐carotene (orange), and zeaxanthin (yellow). After construction of the base strains by metabolic engineering, integration of cell morphology engineering, formation of IMVs and OMVs, and fermentation optimization led to significant improvement in the production of all the seven rainbow colorants. On the basis of previous reports on employing different forms of vesicles for enhancing the production of hydrophobic compounds, we further developed the combined strategies of metabolic and membrane engineering in a systematic and integrative manner. Using the strategies developed here, production of β‐carotene and deoxyviolacein (the two representative natural colorants employed in this study) could be significantly enhanced by 18.4‐fold (Figure 4B,I) and 10.9‐fold (Figure 4G,J), respectively. In particular, the titer of deoxyviolacein (purple colorant) reached more than 11 g L^–1^. In the best β‐carotene producer (BTC1 harboring pWAS‐anti‐*rffDrfaD*), *rffD* and *rfaD* were knocked down for OMV generation, while in the best deoxyviolacein producer (DVIO harboring pWAS‐anti‐*rfaI*‐*cav1*), *rfaI* was knocked down and *cav1* was overexpressed for the simultaneous generation of OMV and IMV. It should be noted that this study focused on applying integrated membrane engineering strategies to a basic metabolically engineered strain to enhance production of natural colorants. Thus, there are more rooms to increase the titers, yields, and productivities of these rainbow colorants by further systems metabolic engineering^[^ ^47^ ^]^ of the strains developed here. Also, similar strategies can be employed for the expansion of colorants spectrum beyond the seven colorants reported here. When it comes to target products that are directly consumed by people such as food or cosmetics, considering the public concerns on the safety of genetically modified organisms (GMOs) is important. In this regard, active and transparent communications on the purified products produced by GMOs between not only engineers and scientists but also policy makers, environmentalists, and the public are required to aptly translate high‐performing microbial cell factories into the industries. Taken together, the metabolic engineering and membrane structure expansion strategies developed here can also be generally employed to develop highly efficient microbial cell factories producing other hydrophobic natural compounds, which will contribute to improving quality of life and resolving environmental issues.

## 4 Experimental Section

##### Materials and Strains

Violacein and deoxyviolacein were purchased from AG Scientific. Lycopene, β‐carotene, canthaxanthin, and astaxanthin were purchased from Merck, and zeaxanthin was purchased from Cayman. All strains used in this study are listed in Table S1, Supporting Information. *E. coli* DH5α (Invitrogen) was used for routine gene cloning works. The previously constructed WLGB‐RPP strain was used as the host strain for carotenoids production,^[^ ^29^ ^]^ and the previously constructed IND5 (pTacGEL) was used as the host strain for the production of violacein derivatives.^[^ ^14^ ^]^ *Chromobacterium violaceum* was provided from Korean Collection for Type Cultures (KCTC), which was used to extract the violacein BGC.

##### Plasmid Construction

Standard protocols were used for PCR, gel electrophoresis, and transformation experiments as were previously reported.^[^ ^48^ ^]^ The plasmids and oligonucleotides used in this study are listed in Tables S1 and S2, Supporting Information, respectively. For routine gene cloning, *E. coli* DH5α containing recombinant plasmids were cultured in Luria‐Bertani (LB) medium or on LB agar plates (1.5%, w/v, agar) at 37 °C supplemented with appropriate concentrations of antibiotics when necessary: 50 µg mL^–1^ of kanamycin (Km), 100 µg mL^–1^ of ampicillin (Ap), and/or 100 µg mL^–1^ of spectinomycin (Spc). Polymerases used for PCR reactions were either Lamp‐Pfu, Pfu (Biofact), or Pfu‐X (Solgent). Restriction endonucleases were purchased from either Enzynomics or New England Biolabs. Either restriction enzyme digestion and Gibson assembly was performed to construct plasmids used in this study.^[^ ^49^ ^]^

To construct the pLYC library, the backbone plasmid pTac15K was linearized by PCR‐amplification using primers pTac15k_F and pTac15k_R. Then, *crtE*, *crtB* and *crtI* genes PCR‐amplified from pCar184^[^ ^29^ ^]^ using primers 16UTR‐crtE_F/crtE_R, 16UTR‐crtB_F/crtB_R, and 16UTR‐crtI_F/crtI_R, respectively, were assembled together with linearized pTac15K by Gibson assembly.^[^ ^49^ ^]^ To construct the pBTC library, the backbone plasmid pTrcCDFS^[^ ^40^ ^]^ was first linearized by PCR‐amplification using pTrcCDF_F/pTrcCDF_R primers. The PCR‐amplified *crtY* from pCar184 using 16UTR‐crtY_F/crtY_R1 primers was then assembled together with linearized pTrcCDFS by Gibson assembly. For the pZEA library construction, PCR‐amplified *crtY* and *crtZ* from pCar184 using 16UTR‐crtY_F/crtY_R2, and 16UTR‐crtZ_F/crtZ_R primers, respectively, were assembled together with linearized pTrcCDFS by Gibson assembly. To construct the pATX library, *trCrBKT* was PCR‐amplified from pAX1^[^ ^12^ ^]^ using 16UTR‐trCrBKT_F/trCrBKT_R primers and cloned into the corresponding site of pZEA which is described in Table S2, Supporting Information.

For the production of violacein derivatives, individual plasmids harboring *vioAB*, *vioC*, *vioD*, *vioCD*, and *vioE* from *C. violaceum* were first constructed. Plasmid pTacCDFS was used as the template for inverse PCR using the primer pair pTacCDFS_inv_F/pTacCDFS_inv_R. Then, the *vioAB* gene fragment was amplified in two split fragments by PCR amplification; the first fragment was amplified using vioAB_F/vioAB_mid_R; the second fragment was amplified using vioAB_mid_F/vioAB_R. The two DNA fragments were assembled with the linearized pTacCDFS plasmid by Gibson assembly to construct the plasmid pTacCDFS‐*vioAB*. Same method was used to construct plasmids pTacCDFS‐*vioC*, pTacCDFS‐*vioD*, pTacCDFS‐*vioCD*, and pTacCDFS‐*vioE*. The gene fragments *vioC*, *vioD*, *vioCD*, and *vioE* were PCR amplified using the primer pairs vioC_F/vioC_F, vioD_F/vioD_R, vioC_F/vioD_R, and vioE_F/vioE_R. Each fragment was assembled with the linearized pTacCDFS plasmid by Gibson assembly to construct the corresponding plasmids. Then, for the production of prodeoxyviolacein, plasmid pPDVIO (pTacCDFS harboring *vioABE*) was constructed as follows. DNA fragment containing *tac* promoter and *vioE* was amplified from the plasmid pTacCDFS‐*vioE* using the primers vioE_frag_F/vioE_frag_R. The amplified gene fragment was assembled with the SacI‐digested plasmid pTacCDFS‐*vioAB* to construct pPDVIO. To produce proviolacein, deoxyviolacin, and violacein, plasmids pPVIO (pTacCDFS harboring *vioABDE*), pDVIO (pTacCDFS harboring *vioABCE*), and pVIO (pTacCDFS harboring *vioABCDE*) were constructed as follows. DNA fragments, each containing *tac* promoter and either of *vioC*, *vioD*, and *vioCD*, were PCR amplified from the plasmids pTacCDFS‐*vioC*, pTacCDFS‐*vioD*, and pTacCDFS‐*vioCD* using the same primers vio_frag_F/vioE_frag_R. The amplified gene fragments were assembled with the SacI‐digested plasmid pTacCDFS‐*vioABE* to construct pTacCDFS‐*vioABCE*, pTacCDFS‐*vioABDE*, and pTacCDFS‐*vioABCDE*.

The *cav1*, *cav2*, and *cav3* genes from *Homo sapiens* were codon optimized and synthesized by Integrated DNA Technologies. To construct pTrc99A‐*cav1*, the synthesized *cav1* gene was inserted into pTrc99A at EcoRI and BamHI sites by Gibson assembly. To construct plasmids pTrc99A‐*cav2* and pTrc99A‐*cav3*, the corresponding genes (*cav2* and *cav3*) were first PCR amplified by using the synthesized gene fragments as templates. The primer BamHIcut_GR was used in common, and cav2_F and cav3_F were used as the other pair of BamHIcut_GR to amplify *cav2* and *cav3*, respectively. The amplified genes were inserted into the pTrc99A plasmid at NcoI and BamHI sites by Gibson assembly. To construct pTrc99A‐*cav12* (harboring *cav1* and *cav2*), the *cav2* gene was PCR amplified using primers cav2_BamHI_F/PstI_HindIII_Gib_R, using pTrc99A‐*cav2* as the template. The amplified gene fragment was inserted into the pTrc99A‐*cav1* plasmid at BamHI and PstI sites using restriction enzyme digestion and ligation. To construct pTrc99A‐*cav23* (harboring *cav2* and *cav3*) and pTrc99A‐*cav13* (harboring *cav1* and *cav3*), the *cav3* gene was PCR amplified using primers cav3_PstI_F/PstI_HindIII_Gib_R. The amplified gene fragment was inserted into pTrc99A‐*cav2* and pTrc99A‐*cav1*, respectively, at PstI site by restriction enzyme digestion and ligation. To construct pTrc99A‐*cav123* (harboring *cav1*, *cav2*, and *cav3*), the abovementioned PCR amplified *cav3* fragment was inserted into pTrc99A‐*cav12* at PstI site by restriction enzyme digestion and ligation.

Construction of pTrc99A‐*plsBC* was performed by inserting the PCR amplified *plsB* and *plsC* gene fragments into the pTrc99A plasmid at PstI and HindIII sites by Gibson assembly. The *plsB* and *plsC* genes were PCR amplified from the genomic DNA of *E. coli* W3110 by using primer pairs plsB_GF/plsB_GR and plsC_GF/plsC_GR, respectively. Then, the *plsBC* gene fragment *was* isolated from the pTrc99A‐*plsBC* plasmid by digesting the plasmid using PstI and HindIII. The *plsBC* gene fragment was inserted into pTrc99A‐*cav1* at PstI and HindIII sites to construct the pTrc99A‐*cav1*‐*plsBC* plasmid.

To test the synergistic effects of introducing multiple sRNAs, plasmids pWAS‐anti‐*rffDrfaD*, pWAS‐anti‐*rffDrfaQ*, pWAS‐anti‐*rfaQD*, pWAS‐anti‐*rfaQI*, and pWAS‐anti‐*mrdBrfaI* were constructed. First, sRNA fragments (anti‐*rfaD*, anti‐*rffD*, anti‐*rfaI* and anti‐*mrdB*) were PCR amplified using primers sRNAdouble_Gib_F/sRNAdouble_Gib_R. Then, anti‐*rfaD*, anti‐*rffD*, and anti‐*rfaI* fragments were inserted into the linearized anti‐*rfaQ* sRNA‐harboring plasmid (linearized by PCR using primers sRNAdouble_IV_F/sRNAdouble_IV_R) by Gibson assembly to construct pWAS‐anti‐*rfaQD*, pWAS‐anti‐*rffDrfaQ*, and pWAS‐anti‐*rfaQI*. The anti‐*rffD* fragment was inserted into the linearized anti‐*rfaD* sRNA‐harboring plasmid (also linearized by PCR using primers sRNAdouble_IV_F and sRNAdouble_IV_R) by Gibson assembly to construct pWAS‐anti‐*rffDrfaD*. To introduce *plsBC* into sRNA‐harboring plasmids, the *plsBC* gene fragment was amplified from pTrc99A‐*plsBC* using primers ptrc‐cav1_GF/ptrc‐plsBC_GR, and was inserted into the corresponding sRNA plasmids (pWAS‐anti‐*rffD*, pWAS‐anti‐*rfaD*, pWAS‐anti‐*rfaI*, and pWAS‐anti‐*rfaQ*) at SphI site. To introduce *cav1* into sRNA‐harboring plasmids, the *cav1* gene fragment was amplified from pTrc99A‐*cav1* using primers ptrc‐cav1_GF/ptrc‐cav1_GR, and was inserted into the corresponding sRNA plasmids (pWAS‐anti‐*mrdB*, pWAS‐anti‐*rfaI*, pWAS‐anti‐*mrdBrfaI*).

To merge *cav1* with the sRNA plasmids, the *cav1* gene fragment was amplified from pTrc99a‐*cav1* using primers ptrc‐cav1_GF/ptrc‐cav1_GR, and was inserted into the sRNA plasmids (pWAS‐anti‐*rffDrfaD*, pWAS‐anti‐*mrdB*, pWAS‐anti‐*rfaI*, and pWAS‐anti‐*mrdBrfaI*) at SphI site, each constructing pWAS‐anti‐*rffDrfaD*‐*cav1*, pWAS‐anti‐*mrdB*‐*cav1*, pWAS‐anti‐*rfaI*‐*cav1*, and pWAS‐anti‐*mrdBrfaI*‐*cav1*. pWAS‐anti‐*rffDrfaD*‐*cav1*‐*plsBC* was constructed by inserting *cav1*‐*plsBC* gene fragment amplified from pTrc99a‐*cav1*‐*plsBC* using ptrc‐cav1_GF/ptrc‐plsBC_GR primers into SphI site of the pWAS‐anti‐*rffDrfaD* plasmid.

##### Media and Culture Conditions

Strains for the production of carotenoids were first inoculated from colonies on LB agar plates into 14 mL test tube containing 3 mL of Terrific Broth (TB) medium (20 g tryptone, 24 g yeast extract, 4 mL of glycerol, 0.017 m KH_2_PO_4_, and 0.072 m K_2_HPO_4_ per liter) supplemented with appropriate antibiotics, and were cultivated at 30 °C, shaken in a rotary shaker at 220 rpm. When OD_600_ reached ≈1–2, 1 mL aliquot of each culture was transferred to 250 mL baffled flasks containing 20 mL of TB medium and cultivated at 30 °C at 200 rpm until OD_600_ reached ≈1–2. Strains for the production of violacein derivatives were first inoculated from colonies on LB agar plates into 25 mL test tube containing 10 mL of LB medium supplemented with appropriate antibiotics, and were cultivated in a rotary shaker at 220 rpm, at 37 °C overnight. For both carotenoids strains and violacein derivatives strains, 1 mL aliquot of each seed culture was transferred to a 250 mL baffled flask containing 50 mL of R/2 medium supplemented with 3 g L^–1^ of yeast extract and 20 g L^–1^ of glycerol (for violacein derivatives, 3 g L^–1^ of (NH_4_)_2_SO_4_ was additionally added) which was incubated at 30 °C and at 200 rpm. The R/2 medium (pH 6.8) contains the followings per liter: 2 g (NH_4_)_2_HPO_4_, 6.75 g KH_2_PO_4_, 0.85 g citric acid, 0.7 g MgSO_4_·7H_2_O, and 5 mL trace metal solution (TMS) **[**10 g FeSO_4_·7H_2_O, 2.25 g ZnSO_4_·7H_2_O, 1 g CuSO_4_·5H_2_O, 0.5 g MnSO_4_·5H_2_O, 0.23 g Na_2_B_4_O_7_·10H_2_O, 2 g CaCl_2_·2H_2_O and 0.1 g (NH_4_)_6_Mo_7_O_24_ per liter of 5 m HCl].^[^ ^40^ ^]^ When OD_600_ of the cultures reached ≈0.6–0.8, 1 mm isopropyl *β*‐D‐1‐thiogalactopyranoside (IPTG) was added to induce gene expression. When required, 50 mg L^–1^ of Km, 100 mg L^–1^ of Ap, and/or 100 mg L^–1^ of Spc was added to the medium. After induction, the cells were cultivated for 36 h for carotenoids and 48 h for violacein derivatives.^[^ ^12^, ^41^ ^]^ Cell growth was monitored by measuring the absorbance at 600 nm (OD_600_) with an Ultrospec 3100 spectrophotometer (Amersham Biosciences). The dry cell weight (DCW) was measured after drying the cell pellets at 75 °C over 48 h.

##### Fed‐Batch Fermentation

Fed‐batch fermentations were conducted in 6.6 L jar fermenters (BioFlo 320, Eppendorf) containing 1.6 L R/2 medium (pH 6.95) supplemented with 30 g L^–1^ glucose or glycerol, 3 g L^–1^ yeast extract, and appropriate antibiotics (for strains producing carotenoids) or 1.95 L R/2 medium (pH 6.8) supplemented with 20 g L^–1^ glucose or glycerol, 3 g L^–1^ yeast extract, 3 g L^–1^ of (NH_4_)_2_SO_4_, and appropriate antibiotics (for strains producing violacein derivatives). For the production of carotenoids, cells were inoculated from a colony into a 14 mL test tube containing 3 mL TB medium supplemented with appropriate antibiotics and were cultivated in a rotary shaker at 30 °C and 220 rpm until OD_600_ reached ≈1–2. The seed culture (1 mL) was transferred into a new 250 mL baffled flask containing 50 mL R/2 medium supplemented with 3 g L^–1^ yeast extract, 20 g L^–1^ glycerol, and appropriate antibiotics. The cells were cultivated at 30 °C and 200 rpm until OD_600_ reached ≈3–4, and were transferred into the fermenter. For the production of violacein derivatives, cells were inoculated from a colony into a 25 mL test tube containing 10 mL LB medium supplemented with appropriate antibiotics and were cultivated in a rotary shaker at 37 °C and 200 rpm, overnight. Then, a 250 mL baffled flask, containing 50 mL of R/2 medium supplemented with 20 g L^–1^ glycerol or glucose, 3 g L^–1^ (NH_4_)_2_SO_4_, 3 g L^–1^ yeast extract, and appropriate antibiotics, was inoculated with 1 mL aliquot of the seed culture. The cells were cultured at 30 °C and 200 rpm until OD_600_ reached ≈4, and were transferred into the fermenter. The culture pH was controlled at 6.8 by automatic feeding of 28% (v/v) ammonia solution, and the temperature was maintained at 30 °C.

In the fed‐batch fermentations for the production of carotenoids and violacein derivatives, the dissolved oxygen concentration (DO) was controlled at 40% of air saturation by supplying air at 2 L min^–1^, automatically increasing the agitation speed up to 1 000 rpm, and changing the percentage of pure oxygen added. When OD_600_ of the culture reached ≈20–30 for carotenoids and ≈20 for violacein derivatives, 1 mm IPTG was added to induce enzyme expression. The pH‐stat feeding strategy was employed in order to supply exhausted nutrients to the fermenter. The feeding solution for the production of carotenoids contains the following per liter: 800 g glucose or 817 g glycerol, 6 mL TMS, and 12 g MgSO_4_·7H_2_O. The feeding solution for the production of violacein derivatives contains the following per liter: 650 g glucose or 800 g glycerol, 85 g (NH_4_)_2_SO_4_, 6 mL TMS, and 8 g MgSO_4_·7H_2_O. When the pH becomes higher than 7 (for carotenoids production) or 6.85 (for violacein derivatives production) due to carbon source exhaustion, the feeding solution was automatically added.

##### Construction of 5′UTR Libraries

5′UTR libraries for *crtE*, *crtB*, *crtI*, *crtY*, *crtZ*, and *trCrBKT* genes were designed using UTR library designer software.^[^ ^28^ ^]^ Template 5′UTR sequence was set as 5′‐NNNNNNNNNNAAAGGAGCATCNNNN‐3′ without any additional constraint, and 16S rRNA sequence was set as the default value (5′‐ACCUCCUUA‐3'). Initial 35 nucleotide sequence of each gene starting from ATG was put in the protein coding sequence box. Desired maximum and minimum expression levels were set at 100 000 and 1 000, respectively, and the number of intermediates was set at 8–16.

##### Electron Microscopy

For TEM, 1 mL of cell culture (using flask culture conditions described in the above section) was washed with distilled water and prefixed in 2% paraformaldehyde‐2% glutaraldehyde mixture buffered with Dulbecco's phosphate‐buffered saline (DPBS) at 4 °C overnight. Next, the cells were postfixed in 1% osmium tetroxide solution buffered with DPBS for 1 h at room temperature (25 °C). The fixed samples were dehydrated in graded ethanol, substituted with propylene oxide, and finally embedded in EMbed‐812 resin. polymerization was performed at 60 °C. Ultrathin‐sectioning of the sample was performed in the EM & Histology Core Facility, at the BioMedical Research Center (BMRC), KAIST. The embedded sample was ultrathin‐sectioned (100 nm) with an Ultracut EM UC7 Ultramicrotome (Leica) installed at the BMRC and double‐stained with uranyl acetate and lead citrate. TEM imaging was performed in the EM & Histology Core Facility at the BMRC or in National Nanofab Center (NNFC), KAIST. The prepared samples were examined under Tecnai G2 Spirit TWIN (FEI) installed at BMRC at 120‐kV acceleration voltage (Figures 2G*–*J and 3D,E) or Tecnai G2 F30 S‐Twin (FEI) installed at NNFC at 300‐kV acceleration voltage (Figure 3I,J).

For SEM, 1 mL of cell culture or vesicles was washed with distilled water and resuspended in distilled water. The sample was dried on a silicon wafer and sputter‐coated with osmium before examination with SU8230 scanning electron microscope (Hitachi) at 2‐kV (Figures 2H,J and 3F) or at 3‐kV (Figures 2G,I and 3D,E,I,J) acceleration voltage installed at KAIST Analysis Center for Research Advancement (KARA).

##### OMV Purification

To purify OMVs for SEM analysis or colorants quantification, cell‐free culture supernatant was collected, and 400 µL of the supernatant was mixed with 200 µL of Total Exosome Isolation Kit (Invitrogen) by vortexing. The mixture was incubated at 4 °C overnight, and was centrifuged at 10 000 × *g* for 20 min at 4 °C. After removing the supernatant, the remaining pellet was resuspended in 100 µL of 1X PBS for SEM analysis or in DMSO for quantification.

##### Analytical Procedures

After culture, cells were harvested by centrifugation at 16 000 × *g* for 1 min. To quantify the production of carotenoids in the cells, metabolites were extracted from each cell pellet with 1 mL of analytical grade acetone and was vortexed vigorously using Thermo shaker (TS100, Ruicheng) for 15 min at 55 °C and 1 500 rpm. The lysed cells were centrifuged at 16 000 × *g* for 1 min to separate the acetone solution containing extracted carotenoids. To quantify total production of violacein derivatives, 50 µL of the cell culture was mixed with 950 µL DMSO and was vortexed using Thermo shaker (TS100, Ruicheng) at 40 °C and 1 500 rpm. To quantify violacein derivatives accumulated in the cell, metabolites were extracted from each cell pellet with 1 mL of DMSO and was vortexed using Thermo shaker (TS100, Ruicheng). The mixture was centrifuged at 16 000 × *g* for 3 min to separate the DMSO solution containing extracted violacein derivatives. Carotenoids secreted to the culture medium were extracted by mixing the cell‐free culture supernatant with 10× volume of acetone using the Thermo shaker at 55 °C and 1 500 rpm for 15 min. Secreted violacein derivatives were extracted by mixing the cell‐free culture supernatant with equal volume of ethyl acetate, vortexing using the Thermo shaker at 40 °C and 1 500 rpm, and separating the organic layer. The prepared samples were analyzed with HPLC (1260 Infinity II; Agilent) equipped with DAD detectors (G7115A; Agilent). YMC carotenoid C_30_ column (YMC) was used for carotenoids analysis and Eclipse XDB‐C18 column (4.6 × 150 mm; Agilent) was used for violacein derivatives analysis. For the analysis of carotenoids, mobile phase was run at a flow rate of 0.6 mL min^–1^; the mobile phase consists of solvent A [90% (v/v) methanol in distilled water] and solvent B (tert‐butyl methyl ether). The following gradient was applied: 0–3 min, an isocratic condition at 10% solvent B; 3–15 min, a linear gradient of solvent B from 10% to 70%; 15–20 min, a linear gradient of solvent B from 70% to 90%; 20–29 min, an isocratic condition at 90% solvent B; 29–30 min, a linear gradient of solvent B from 90% to 10% (all in vol%). For the analysis of violacein derivatives, mobile phase was run at a flow rate of 1 mL min^–1^; the mobile phase consists of solvent A [0.1% (v/v) formic acid in distilled water] and solvent B (acetonitrile). The following gradient was applied: 0–3 min, an isocratic condition at 10% solvent B; 3–10 min, a linear gradient of solvent B from 10% to 100%; 10–15 min, an isocratic condition at 100% solvent B (all in vol%). Samples were monitored at 450 nm for carotenoids and 570 nm for violacein derivatives. Concentration of each product was determined by mapping the area of HPLC peaks to each calibration curve generated using dilutions of authentic standard chemicals. For screening initially cultured strains producing carotenoids, extracted samples were analyzed by measuring absorbances at 474, 453, 452, and 475 nm, respectively for lycopene, β‐carotene, zeaxanthin, and astaxanthin, using an Ultrospec 3100 spectrophotometer (Amersham Biosciences).

As commercial standards were not available for proviolacein and prodeoxyviolacein, the violacein derivatives produced from engineered *E. coli* strains were further analyzed through HPLC (1100 Series HPLC; Agilent) connected with MS (LC/MSD VL; Agilent). Eclipse XDB‐C18 column (4.6 × 150 mm; Agilent) was used and operated at 25 °C. Two mobile phase solvents were used: solvent A (0.1% formic acid) and solvent B (0.1% formic acid in acetonitrile). The total flow rate was maintained at 0.6 mL min^–1^, and the following gradient was applied: 0–1 min, 30% solvent B; 1–10 min, a linear gradient of solvent B from 30% to 70%; 10–20 min, 70% solvent B (all in vol%). The eluent was continuously injected into the mass spectrometry using electrospray ionization positive ion mode with the following conditions: fragmentor, 180 V; drying gas flow, 12.0 L min^–1^; drying gas temperature, 350 °C; nebulizer pressure, 30 psig; capillary voltage, 2.5 kV. For analysis, scan mode was used and the scanned mass range was *m*/*z* of 200–400. Also, proviolacein and prodeoxyviolacein were purified via fraction collector (G1364C; Agilent) attached to a HPLC (1260 Infinity II; Agilent) equipped with DAD detectors (G7115A; Agilent). Eclipse XDB‐C18 column (4.6 × 150 mm; Agilent) was used. Mobile phase was run at a flow rate of 0.6 mL min^−1^; the mobile phase consists of solvent A [0.1% (v/v) formic acid in distilled water] and solvent B (acetonitrile). The following gradient was applied: 0–1 min, an isocratic condition at 30% solvent B; 1–10 min, a linear gradient of solvent B from 30% to 70%; 10–20 min, a linear gradient of solvent B from 70% to 95% (all in vol%). The concentrations of natural colorants were determined by mapping the area of HPLC peaks to each calibration curve generated using the standard chemicals.

Absorption spectra of colorants extracted from cell cultures were obtained by step‐scanning of absorbance at 2 nm intervals in the wavelength range from 350 to 700 nm using Multimode Microplate Reader (Tecan).

##### Statistical Analysis

Sample sizes were not predetermined. All colonies were randomly selected from plates containing ≈100–200 colonies and subject to independent flask culture and chemical analysis. All numerical data are presented as mean ± SD (standard deviation) from experiments done in triplicates. Means were compared using a two‐tailed Student's *t*‐test.^[^ ^50^ ^]^ *P* values were obtained by either Microsoft Excel 2016 or OriginPro 2019. *P* values are represented as *P < 0.05, **P < 0.01 or ***P < 0.001, which are considered as significant. When multiple hypotheses were tested, the significance level thresholds were divided by the number of hypotheses, according to Bonferroni correction (corrected significance levels represented as *α*/*m*; *α*, original significance level; *m*, number of hypotheses). Thus, *P < 0.05/*m*, **P < 0.01/*m* or ***P < 0.001/*m*. The investigators were blinded to the group allocation by randomly selecting single colonies multiple times.

## Conflict of Interest

D.Y., S.Y.P., and S.Y.L. declare that the membrane engineering technologies described here are patent filed including, but not limited to KR 10‐2020‐0144521.

## Author Contributions

D.Y. and S.Y.P. contributed equally to this work. S.Y.L. conceived the project, D.Y., S.Y.P., and S.Y.L. designed the experiments. D.Y. and S.Y.P. conducted the experiments and analyzed the data. D.Y., S.Y.P., and S.Y.L. wrote the manuscript. All authors read and approved the final manuscript.

## Supporting information

Supporting Information

Supplemental Movie 1

Supplemental Movie 2

## Data Availability

All datasets analyzed during the current study are presented in this manuscript, or are available from the corresponding author upon reasonable request.
