# Supplementary material for: Production of Rainbow Colorants by Metabolically Engineered Escherichia coli
Source: Adv Sci (Weinh). 2021 May 25;8(13):2100743. doi: 10.1002/advs.202100743 (PMC8261500; doi:10.1002/advs.202100743)
Supplement: Supplementary file 1 — Supporting Information [file ADVS-8-2100743-s001.pdf]

© 2021 Wiley-VCH GmbH

ADVANCED SCIENCE
Open Access

Supporting Information

for *Adv. Sci.*, DOI: 10.1002/advs.202100743

Production of rainbow colorants in Escherichia coli

Dongsoo Yang, Seon Young Park, and Sang Yup Lee*

Supporting Information

Production of rainbow colorants in *Escherichia coli*

*Dongsoo Yang, Seon Young Park, and Sang Yup Lee**

Email: leesy@kaist.ac.kr
D.Y. and S.Y.P. contributed equally to this work.

This PDF file includes:

Texts S1 to S3
Figs. S1 to S5
Tables S1 to S5
References for SI reference citations

Other supplementary materials for this manuscript include the following:

Movies S1 to S2

Supporting Information Texts

Text S1. Construction of carotenoids producing strains.

First, expression levels of *crtE*, *crtB* and *crtI* were optimized for enhanced lycopene production. The expression level of each gene was diversified using different 5' UTR sequences designed using UTR library designer software. For each gene, 16 different 5'UTR sequences were designed, and all the three genes paired with corresponding 5'UTR sequences were cloned into pTac15K to generate the pLYC library (Figure S1A). The constructed library was transformed into WLGB-RPP, and 29,000 colonies (more than 5 times the size of the library) were screened. Among them, 200 colonies exhibiting deeper red color were selected and cultured in test tubes (Figure S1B). The top 10 strains from the test tube-scale culture were selected for flask culture for more accurate measurement of the lycopene titers (Figure S1C). In order to compare the performance of the initially selected

strains, absorbance of the culture extracts at 474 nm (A474) was used to compare lycopene titers. As a result, LYC79 produced the highest lycopene titer of 23.9 mg l^{-1} by flask culture and was selected as the base strain for further engineering.

Next, downstream genes required for β-carotene, zeaxanthin, and astaxanthin production from lycopene were sequentially cloned into the pTrcCDFS plasmid. The *crtY* gene was first cloned for β-carotene production, followed by insertion of the *crtZ* gene downstream of *crtY* for zeaxanthin production. For astaxanthin production, the *trCrBKT* gene was inserted downstream of *crtZ*. In order to balance metabolic fluxes, 16 different 5'UTR sequences for each gene were also designed and examined, in the same manner as employed for lycopene production (Figure S1A).

The constructed pBTC (pTrcCDFS harboring *crtY*), pZEA (pTrcCDFS harboring *crtY* and *crtZ*) and pATX (pTrcCDFS harboring *crtY*, *crtZ*, and *trCrBKT*) libraries were respectively transformed into the LYC79 strain and 200, 4,000, and 21,600 colonies (more than 5 times the size of each library) were screened, respectively. Twenty β-carotene producing colonies exhibiting deeper orange colors and 40 zeaxanthin producing colonies exhibiting deeper yellow colors were selected and were cultured in test tubes. The concentrations of β-carotene and zeaxanthin were compared by measuring the absorbance of cell extracts at 473 nm (A473) and 452 nm (A452), respectively (Figure S1D,E). Whereas β-carotene and zeaxanthin overproducing strains could be easily distinguished by their independent orange and yellow colors, respectively, astaxanthin overproducing strains were difficult to be selected since astaxanthin and canthaxanthin (an astaxanthin precursor) both show red color. Therefore, after comparing 200 red colonies by measuring the absorbance of the cell extracts at 475 nm (A475) (Figure S1F), the cell extracts of the top 50 strains were

analyzed again by HPLC to accurately measure the concentrations of astaxanthin (Figure S1G).

The β-carotene, zeaxanthin, and astaxanthin producers selected above were flask cultured for more accurate comparison of strain performance. Ultimately, BTC1 producing 18.7 mg l^{-1} of β-carotene, ZEA20 producing 12.7 mg l^{-1} of zeaxanthin and ATX68 producing 14.5 mg l^{-1} of astaxanthin were selected as the chassis strains for carotenoids production (Figure S1H-J). The 5'UTR library sequences and 5'UTR sequences from the selected strains are listed in Table S3.

Text S2. Sequences of the artificially synthesized genes.

cav1:

ATGTCTGGGGGCAAATACGTAGACTCGGAGGGACATCTCTACACCGTTCCCATCC
GGGAACAGGGCAACATCTACAAGCCCAACAACAAGGCCATGGCAGACGAGCTG
AGCGAGAAGCAAGTGTACGACGCGCACACCAAGGAGATCGACCTGGTCAACCGC
GACCCTAAACACCTCAACGATGACGTGGTCAAGATTGACTTTGAAGATGTGATTG
CAGAACCAGAAGGGACACACAGTTTTCACGGCATTTGGAAGGCCAGCTTCACCA
CCTTCACTGTGACGAAATACTGGTTTTACCGCTTGCTGTCTGCCCTCTTTGGCATC
CCGATGGCACTCATCTGGGGCATTTACTTCGCCATTCTCTCTTTCCTGCACATCTG
GGCAGTTGTACCATGCATTAAGAGCTTCCTGATTGAGATTCAGTGCACCAGCCGT
GTCTATTCCATCTACGTCCACACCGTCTGTGACCCACTCTTTGAAGCTGTTGGGA
AAATATTCAGCAATGTCCGCATCAACTTGCAGAAAGAAATATAA

cav2:

ATGGGGCTTGAGACTGAGAAGGCAGATGTCCAACTGTTCATGGATGATGATTCTT
ACTCACATCACTCAGGACTGGAATATGCAGATCCAGAAAAGTTTGCGGACTCCG
ACCAGGATCGTGACCCCCACCGCTTAAATAGTCACTTAAAACTGGGCTTTGAAGA
TGTGATCGCGGAGCCTGTCACAACTCATAGTTTCGATAAGGTTTGGATTTGCTCA
CACGCATTATTTGAAATTTCAAAGTACGTTATGTATAAGTTCCTTACTGTATTTTT
GGCCATCCCTCTTGCCTTTATCGCAGGAATCCTGTTCGCTACCTTGAGTTGTCTGC
ACATTTGGATTCTTATGCCATTCGTAAAGACATGCCTTATGGTGTTGCCATCAGTG
CAAACCATCTGGAAGTCCGTCACTGATGTAATTATTGCCCCTTTGTGTACATCTGT
GGGCCGCTGCTTTTCGAGCGTCTCACTTCAATTGTCGCAGGATTAA

cav3:

ATGATGGCCGAAGAGCATACCGATCTTGAAGCTCAAATTGTAAAGGATATTCATT
GTAAGGAAATTGACTTGGTTAATCGTGATCCTAAGAACATCAACGAGGATATCGT
TAAGGTAGACTTCGAGGATGTTATTGCAGAACCTGTTGGAACATACAGTTTCGAC
GGTGTCTGGAAGGTGTCGTACACTACGTTTACCGTTAGTAAGTATTGGTGCTATC
GCTTACTGTCCACTCTGTTGGGTGTCCCCCTTGCTTTGCTTTGGGGATTCCTGTTC
GCGTGTATCTCTTTTTGCCATATTTGGGCTGTCGTTCCATGTATTAAATCGTACTT
AATTGAGATTCAATGTATCTCTCATATTTATAGTCTTTGTATCCGCACGTTCTGTA
ATCCCCTTTTTGCGGCCTTGGGGCAGGTGTGCTCAAGTATTAAGGTTGTACTTCGT
AAGGAGGTCTAA

Text S3. Optimization of fermentation conditions for β-carotene and deoxyviolacein production.

For β-carotene production, fed-batch fermentation using the BTC1 (pWAS-anti-*rffDrfaD*) strain was carried out using glucose or glycerol as a carbon source. As a result, higher concentration of β-carotene (343 mg l^{-1}) was produced from glycerol than that (295 mg l^{-1}) from glucose (Figure 4B; Figure S5A-C). For deoxyviolacein production, fed-batch fermentation of the DVIO (pWAS-anti-*rfaI-cav1*) strain was also carried out using glucose or glycerol as a carbon source. In contrast to the case of β-carotene, higher concentration of deoxyviolacein (10.8 g l^{-1}) was produced from glucose than that (6.81 g l^{-1}) from glycerol (Figure 4G; Figure S5F-H). When glucose was used as a sole carbon source, the cell growth at the point of maximum deoxyviolacein production was almost two-fold higher [21.2 g dry cell weight (gDCW) per liter] than that (12.0 gDCW l^{-1}) obtained using glycerol. After fed-batch culture of the DVIO (pWAS-anti-*rfaI-cav1*) strain, we could see a significant amount of aggregates attached on the inner surface of the bioreactor (i.e., glass wall, impeller, metal shaft) consisting OMVs, deoxyviolacein crystals, and cell debris (Figure S5L). As deoxyviolacein contained in these aggregates is not included in samples taken during fermentation, measuring the concentrations of metabolites contained in these aggregates was required. Thus, the aggregates were dissolved in ethanol (of the same volume with the end volume of the fermentation broth) after discarding the cell culture and gently rinsing the inner

surface of the fermenter with water to leave only the aggregates attached to the inner surface. The sum of the deoxyviolacein titer obtained from direct sampling (10.8 g l^{-1}) and that obtained from the aggregates (0.530 g l^{-1}) was employed to represent the total deoxyviolacein titer (11.3 g l^{-1}). The concentrations of other violacein derivatives contained in the aggregates formed on the fermenter inner surface were also measured. All independently performed fed-batch fermentations showed reproducibility (Figure S5).

Supporting Information Figures

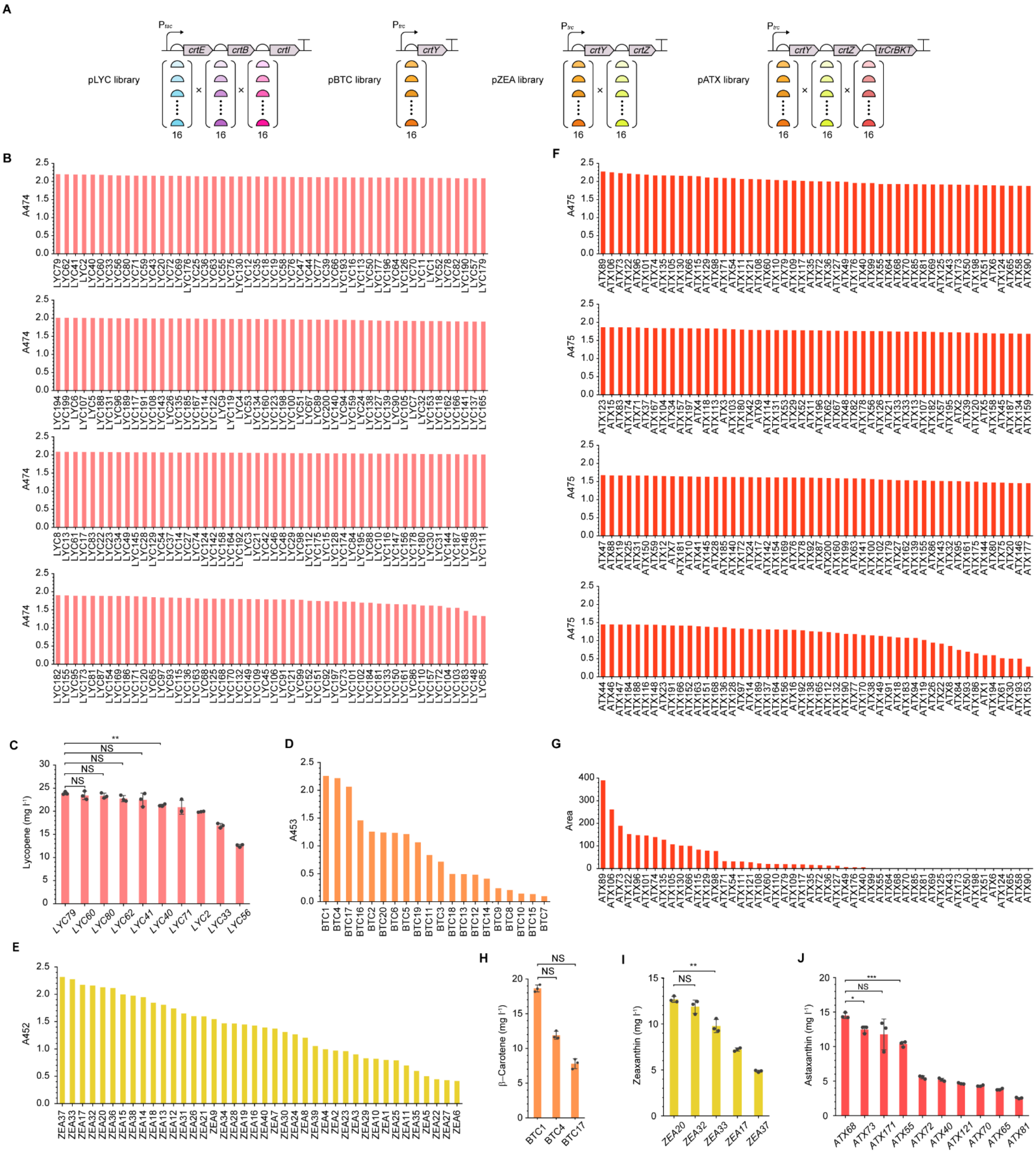

Figure S1. Construction of strains for the production of rainbow colorants. A) Genetic configurations of pLYC, pBTC, pZEA and pATX libraries. Bent arrow, hemisphere, and T-shape denote promoter, 5'UTR, and terminator, respectively. B) Initial screening results of the LYC strains. Colonies exhibiting deeper red colors were cultured in test tubes, and high performing strains were flask cultured as shown in C). $^{*}P < 0.01$, $^{**}P < 0.002$, $^{***}P < 0.0002$, determined by two-tailed Student's *t*-test. (D-F) correspond to test tube screening results of BTC, ZEA and ATX strains, respectively. G) HPLC analysis results of the extracted samples of the top 50 strains selected from (F). The Y-axis denotes the area of astaxanthin peaks. (H), (I), and (J) are flask culture results of selected BTC, ZEA and ATX strains, respectively. (H-I) $^{*}P < 0.025$, $^{**}P < 0.005$, $^{***}P < 0.0005$, determined by two-tailed Student's *t*-test. (J) $^{*}P < 0.017$, $^{**}P < 0.0033$, $^{***}P < 0.00033$, determined by two-tailed Student's *t*-test. Error bars, mean ± SD ($n = 3$). *P*-value thresholds were adjusted using Bonferroni correction (corrected significance levels represented as α/m; α, original significance level; m, number of hypotheses). NS, not significant.

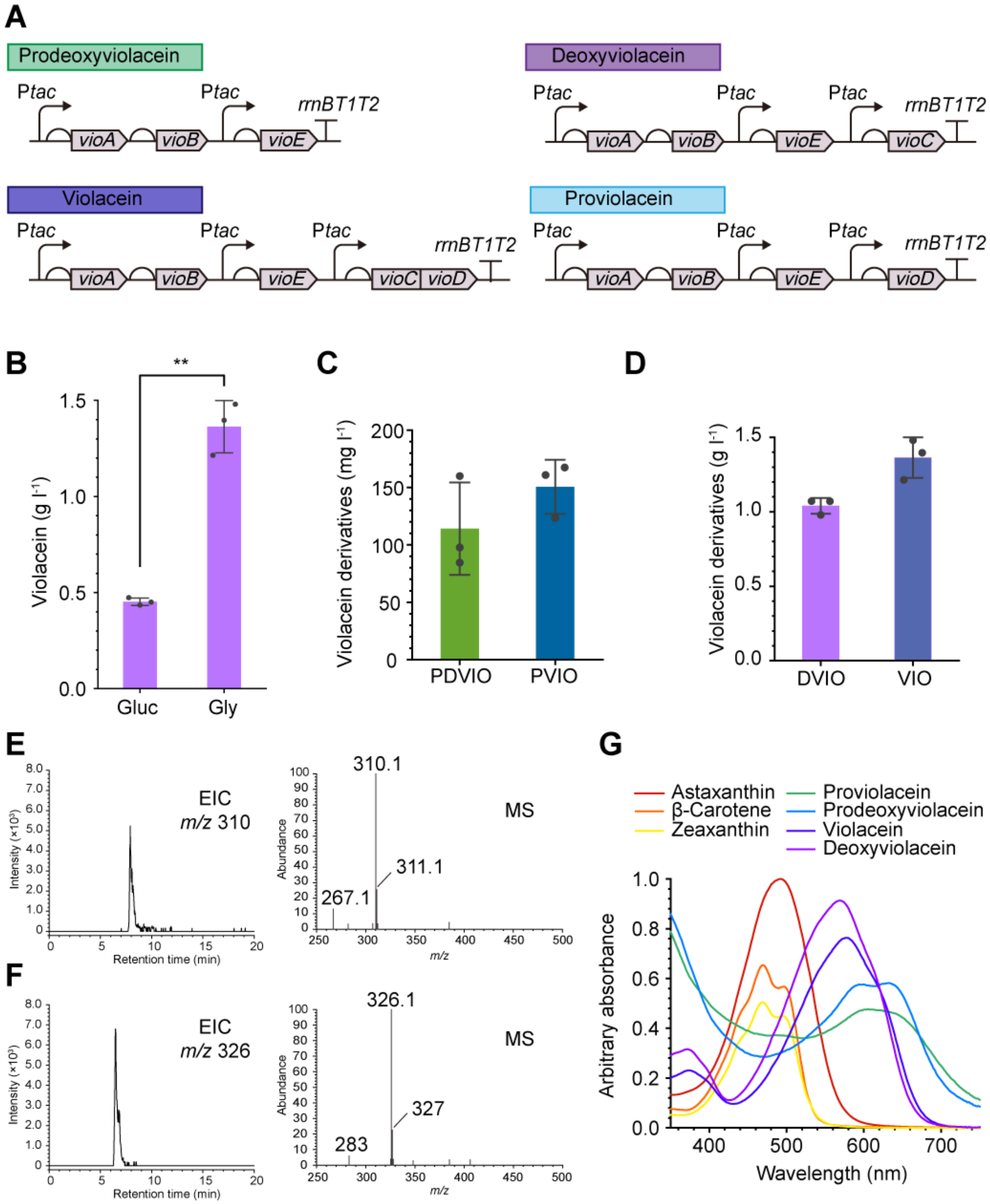

Figure S2. Construction of base strains for the production of violacein derivatives. A) Configurations of plasmids for the production of each violacein derivative. B) Comparison of violacein production by using glucose or glycerol as a sole carbon source. $^{**}P < 0.01$, determined by two-tailed Student's *t*-test. C) Production of prodeoxyviolacein and proviolacein from the PDVIO and PVIO strains, respectively, using glycerol. D) Production of deoxyviolacein and violacein from the DVIO and VIO strains, respectively, using glycerol. LC-MS chromatogram and MS spectrum of (E) prodeoxyviolacein and (F) proviolacein produced from engineered *E. coli* strains. G) Absorption spectrum of the seven rainbow colorants produced by engineered *E. coli* strains. Wavelength ranges from 350 nm to 750 nm. Each data point represents mean value of three individually prepared samples; smooth curves resulted from linking the data points.

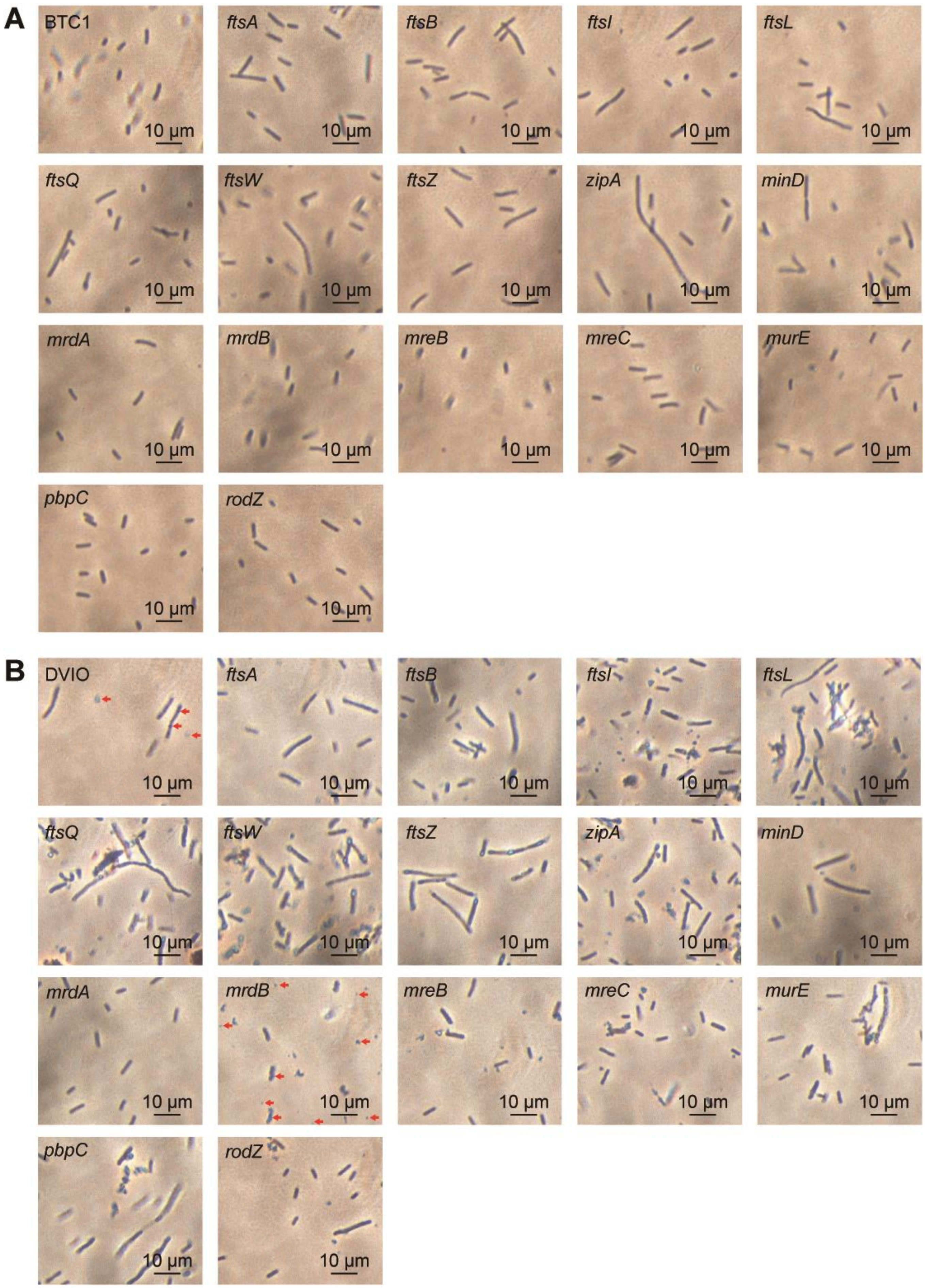
A
BTC1
ftsA
ftsB
ftsI
ftsL
ftsQ
ftsW
ftsZ
zipA
minD
mrdA
mrdB
mreB
mreC
murE
pbpC
rodZ
10 µm
B
DVIO
ftsA
ftsB
ftsI
ftsL
ftsQ
ftsW
ftsZ
zipA
minD
mrdA
mrdB
mreB
mreC
murE
pbpC
rodZ
10 µm

Figure S3. Micrographs of morphology-modified BTC1 and DVIO strains. A) Micrographs of BTC1 harboring cell morphology-related sRNAs. B) Micrographs of DVIO harboring cell morphology-related sRNAs. BTC1 and DVIO denote control strains without sRNAs. The corresponding knockdown target genes are noted on each micrograph. Red arrows indicate deoxyviolacein crystals.

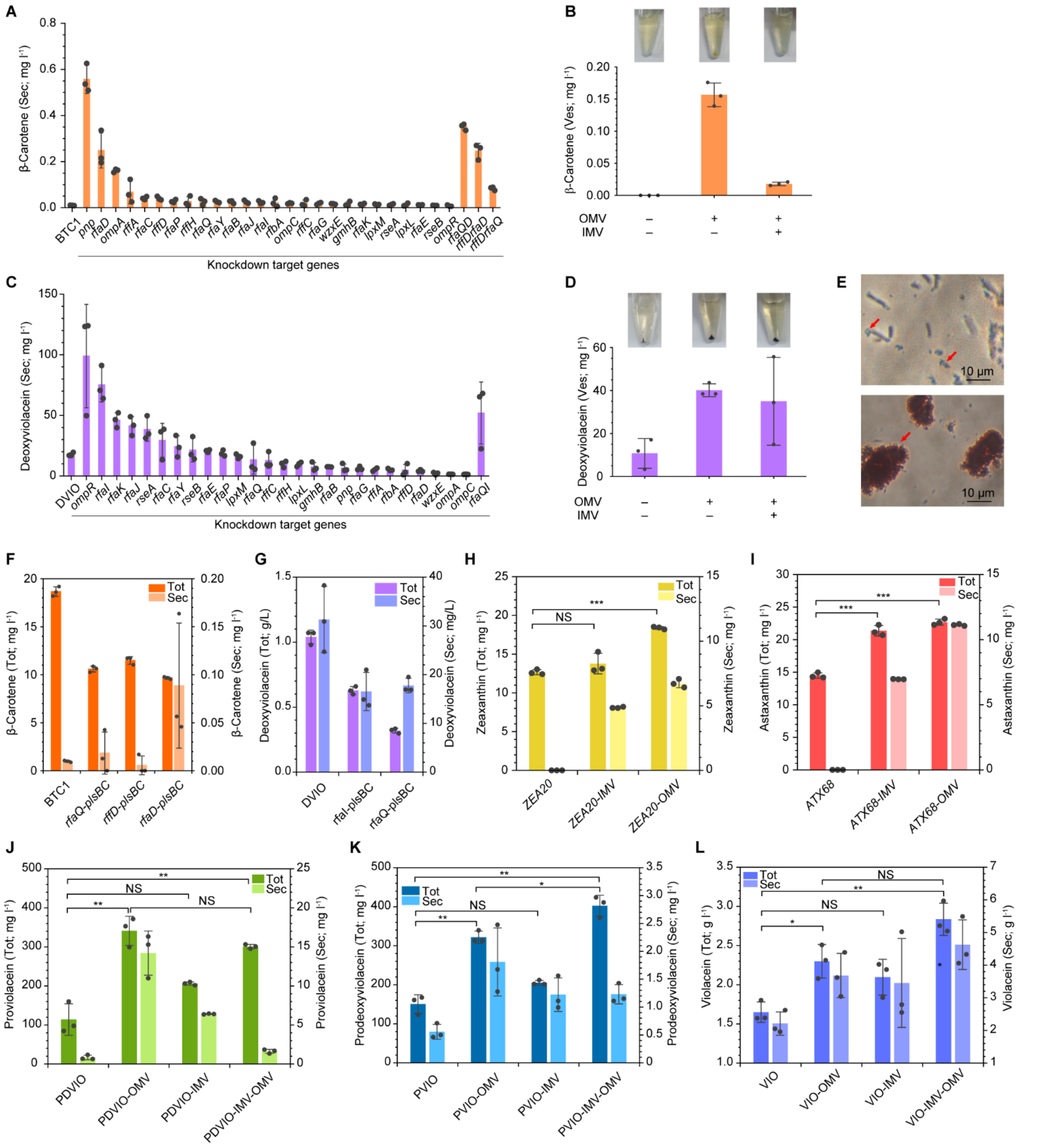

Figure S4. Concentrations of rainbow colorants produced by employing the OMV-forming strategy. A) Concentrations of β-carotene obtained from the extracellular medium after culturing BTC1 harboring sRNAs for OMV formation. B) Concentrations of β-carotene obtained from the purified OMVs. OMV in the X-axis denotes BTC1 (pWAS-anti-*rffDrfaD*); IMV denotes BTC1 (pTrc99A-*cav1-plsBC*). Ves, titers obtained from the purified OMVs. C) Concentrations of deoxyviolacein obtained from the extracellular medium after culturing DVIO harboring sRNAs for OMV formation. D) Concentrations of deoxyviolacein obtained from the purified OMVs. OMV in the X-axis denotes DVIO (pWAS-anti-*rfaI*); IMV denotes DVIO (pTrc99A-*cav1*). Ves, titers obtained from purified OMVs. E) Microscopy photo of DVIO (pWAS-anti-*rfaI*) (upper panel) and aggregates comprising deoxyviolacein crystals, cell debris, and OMVs remained in flasks after rinsing with water (lower panel). Red arrows denote putative OMV aggregates/deoxyviolacein crystals. Production of (F) β-carotene and (G) deoxyviolacein by expression of *plsBC* in addition to sRNAs for OMV formation. Total production (denoted as Tot) and secretory production (denoted as Sec) of (H) zeaxanthin; (I) astaxanthin; (J) prodeoxyviolacein; (K) proviolacein; (L) violacein. (H-I) *$P < 0.025$, **$P < 0.005$, ***$P < 0.0005$, determined by two-tailed Student's t-test. (J-L) *$P < 0.0125$, **$P < 0.0025$, ***$P < 0.00025$, determined by two-tailed Student's t-test. Error bars, mean ± SD ($n = 3$). *$P < 0.05$, **$P < 0.01$, ***$P < 0.001$, NS (not significant) $P \geq 0.05$, determined by two-tailed Student's t-test. P-value thresholds were adjusted using Bonferroni correction (corrected significance levels represented as α/m; α, original significance level; m, number of hypotheses). NS, not significant.

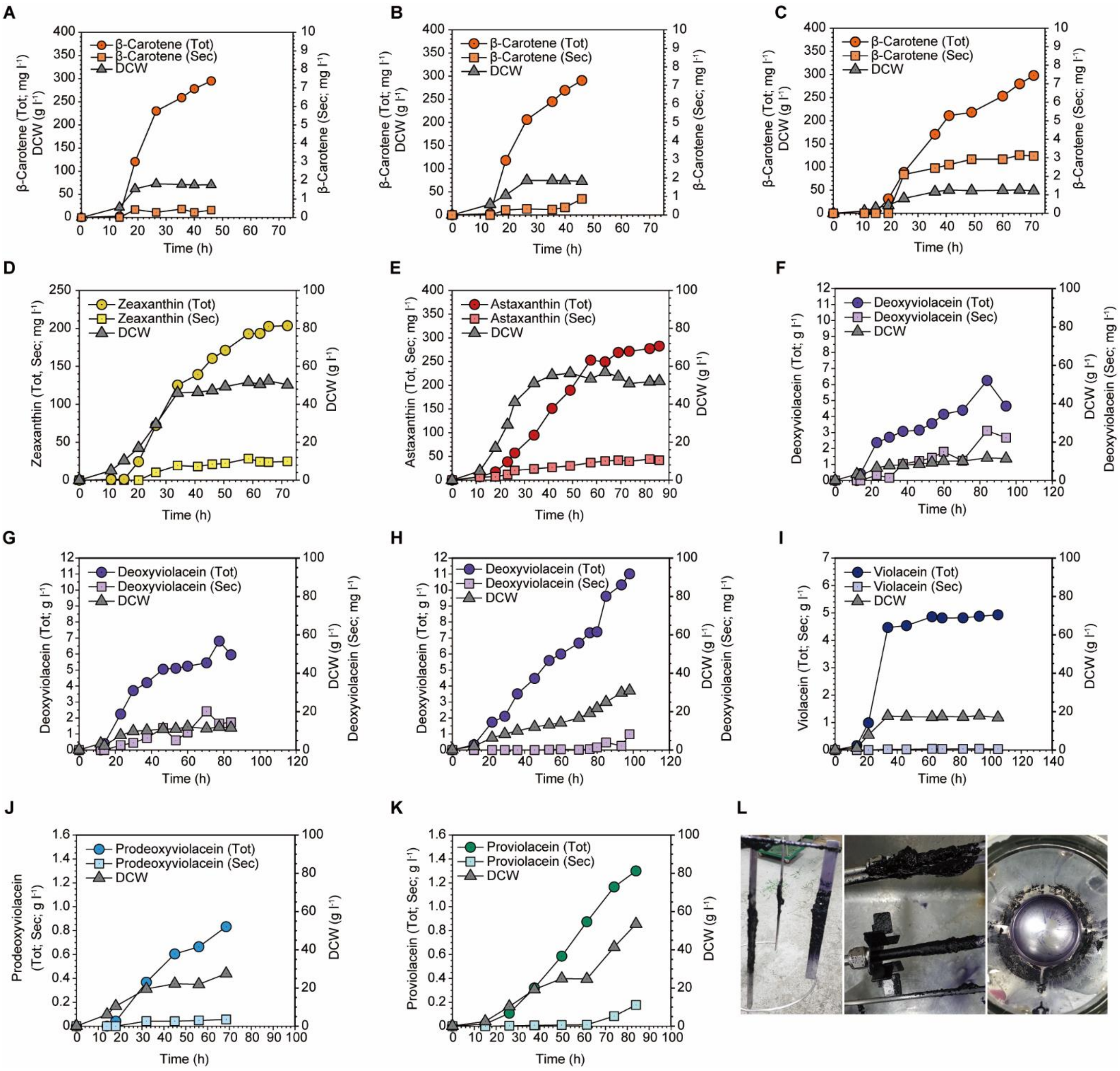

Figure S5. Fed-batch fermentation results of strains producing rainbow colorants. A) Fed-batch fermentation profile of BTC1 (pWAS-anti-*rffDrfaD*) producing β-carotene from glucose and (B) the profile of independently repeated fed-batch fermentation. (C), (D), and (E) each corresponds to the profile of independently repeated fed-batch fermentation for producing β-carotene using BTC1 (pWAS-anti-*rffDrfaD*), zeaxanthin using ZEA20 (pWAS-anti-*rffDrfaD*), and astaxanthin using ATX68 (pWAS-anti-*rffDrfaD*) from glycerol, respectively. F) Fed-batch fermentation profile of DVIO (pWAS-anti-*rfaI-cav1*) producing deoxyviolacein from glycerol and (G) the profile of independently repeated fed-batch fermentation. (H), (I), (J), and (K) each corresponds to the profile of independently repeated fed-batch fermentation for producing deoxyviolacein using DVIO (pWAS-anti-*rfaI-cav1*), violacein using VIO (pWAS-anti-*rfaI-cav1*; 0.80 g l^{-1} of deoxyviolacein was also produced), prodeoxyviolacein using PDVIO (pWAS-anti-*rfaI*), and proviolacein using PVIO (pWAS-anti-*rfaI-cav1*) from glucose. L) Pictures of aggregated OMVs, cell debris, and deoxyviolacein crystals on baffles, impeller and

shafts, and bottom of the fermenter, respecitvely. All independently performed fed-batch fermentations showed reproducibility.

Table S1. *E. coli* strains and plasmids used in this study

***E. coli* strain**	**Description**	**Source**
DH5α	F^- φ80*lacZ*ΔM15 Δ(*lacZYA-argF*)U169 *recA1 endA1 hsdR17*(r_K^- m_K^+) *phoA* Δ*supE44 thi-1 gyrA96 relA1 λ-*	Invitrogen
W3110	F^- *IN(rrnD rrnE)1 rph-1* λ^-	CGSC
WLGB-RPP	W3110 (Δ*lacI* Δ*gdhA* Δ*gpmB ptrc dxs idi ispA pps*)	(1)
IND5	W3110 Δ*trpR* Δ*pykF* Δ*pykA* P*tktA*::P*trc*	(2)
LYC79	WLGB-RPP harboring pLYC79	This study
BTC1	WLGB-RPP harboring pLYC79 and pBTC1	This study
ZEA20	WLGB-RPP harboring pLYC79 and pZEA20	This study
ATX68	WLGB-RPP harboring pLYC79 and pATX68	This study
PDVIO	IND5 harboring pTacGEL and pPDVIO	This study
PVIO	IND5 harboring pTacGEL and pPVIO	This study
DVIO	IND5 harboring pTacGEL and pDVIO	This study
VIO	IND5 harboring pTacGEL and pVIO	This study
**Plasmid**	**Description [a)]**	**Source**
pWAS	Ap^R, ColE1 origin, synthetic sRNA expression vector containing *araC*, *cI*^{ts2} and synthetic sRNA expression cassette (P_R promoter – *micC* – *T1TE*$_T$)	(9)
pTac15K	Km^R, *tac* promoter, P15A origin	(10)
pTrcCDFS	Spc^R, *trc* promoter, CloDF13 (CDF) origin	(5)
pTacCDFS	Spc^R, *tac* promoter, CDF origin	(5)
pTrc99A	Ap^R, *trc* promoter, pBR322 ori	(10)
pTacGEL	Km^R, ColE1 origin, P*tac-aroG*^{fbr}-P*tac-trpE*^{fbr}-P*tac-aroL-rrnBT1T2*$_T$	(2)
pLYC	Km^R, pTac15k derivative, *crtE*, *crtB* and *crtI*, each with 16 different 5'UTR sequences, are cloned at the downstream of the *tac* promoter	This study
pBTC	Spc^R, pTrcCDF derivative, *crtY* with 16 different 5'UTR sequences is cloned at the downstream of the *tac* promoter	This study
pZEA	Spc^R, pTrcCDF derivative, *crtY* and *crtZ*, each with 16 different 5'UTR sequences, are cloned at the downstream of the *tac* promoter	This study
pATX	Spc^R, pZEA derivative, *trCrBKT* with 16 different 5'UTR sequences is cloned at SalI and HindIII sites	This study
pPDVIO	pTacCDF derivative harboring *vioABE*	This study
pPVIO	pTacCDF derivative harboring *vioABDE*	This study
pDVIO	pTacCDF derivative harboring *vioABCE*	This study
pVIO	pTacCDF derivative harboring *vioABCDE*	This study
pTrc99A-*cav1*	pTrc99A derivative harboring *cav1*	This study
pTrc99A-*cav2*	pTrc99A derivative harboring *cav2*	This study
pTrc99A-*cav3*	pTrc99A derivative harboring *cav3*	This study
pTrc99A-*cav12*	pTrc99A derivative harboring *cav1* and *cav2*	This study
pTrc99A-*cav23*	pTrc99A derivative harboring *cav2* and *cav3*	This study
pTrc99A-*cav13*	pTrc99A derivative harboring *cav1* and *cav3*	This study
pTrc99A-*cav123*	pTrc99A derivative harboring *cav1*, *cav2*, and *cav3*	This study
pTrc99A-*cav1-plsBC*	pTrc99A derivative harboring *cav1* and *plsBC*	This study
pWAS-anti-*rffDrfaD*	pWAS derivative containing anti-*rffD* and anti-*rfaD* sRNAs	This study
pWAS-anti-*rffDrfaQ*	pWAS derivative containing anti-*rffD* and anti-*rfaQ* sRNAs	This study
pWAS-anti-*rfaQD*	pWAS derivative containing anti-*rfaQ* and anti-*rfaD* sRNAs	This study
pWAS-anti-*rfaQI*	pWAS derivative containing anti-*rfaQ* and anti-*rfaI* sRNAs	This study
pWAS-anti-*mrdBrfaI*	pWAS derivative containing anti-*mrdB* and anti-*rfaI* sRNAs	This study
pWAS-anti-*rffD-plsBC*	pWAS derivative containing anti-*rffD* sRNA and *plsBC*	This study

pWAS-anti-*rfaD-plsBC*	pWAS derivative containing anti-*rfaD* sRNA and *plsBC*	This study
pWAS-anti-*rfaI-plsBC*	pWAS derivative containing anti-*rfaI* sRNA and *plsBC*	This study
pWAS-anti-*rfaQ-plsBC*	pWAS derivative containing anti-*rfaQ* sRNA and *plsBC*	This study
pWAS-anti-*rffDrfaD-cav1*	pWAS derivative containing anti-*rffD* and anti-*rfaD* sRNAs, and *cav1*	This study
pWAS-anti-*rffDrfaD-cav1-plsBC*	pWAS derivative containing anti-*rffD* and anti-*rfaD* sRNAs, and *cav1* and *plsBC*	This study
pWAS-anti-*mrdB-cav1*	pWAS derivative containing anti-*mrdB* sRNA and *cav1*	This study
pWAS-anti-*rfaI-cav1*	pWAS derivative containing anti-*rfaI* sRNA and *cav1*	This study
pWAS-anti-*mrdBrfaI-cav1*	pWAS derivative containing anti-*mrdB* and anti-*rfaI* sRNAs and *cav1*	This study

[a] Abbreviations: Ap, ampicillin; Km, kanamycin; Spc, Spectinomycin; and R, resistance.

Table S2. Oligonucleotides used in this study

**Primer**	**Sequence (5'-3')** [a]
pTac15K_F	CTTGGCTGTTTTGGCGGATG
pTac15K_R	TGTTTCCTGTGTGAAATTGTTATCCGCTC
16UTR-crtE_F	ATAACAATTTCACACAGGAAACACGYTCMGCGGAAAGRAGCATCGWCCATGTATCCGTTTATAAGGACA
crtE_R	TTAACTGACGGCAGCGAGTT
16UTR-crtB_F	CGCTGCCGTCAGTTAAARCCTTGTTCAAAGGMSYATCTAGGATGAATAATCCGTCGTTACT
crtB_R	TTCGAACGGTTCTTAGAGCGGGCGCTGCCA
16UTR-crtI_F	GCTCTAAGAACCGTTCGAAWGSAGCRTMCAAGATGAAACCAACTACGGTAAT
crtI_R	CGCCAAAACAGCCAAGTTAAATCAGATCCTCCAGC
pTrcCDFS_F	TCTAGAGTCGACCTGCAG
pTrcCDFS_R	TCTGTTTCCTGTGTGAAATT
16UTR-crtY_F	CAATTTCACACAGGAAACAGACCTTCCTCCAWAAGRAGCATCMASTATGGGAGCGGCTATG
crtY_R1	GCAGGTCGACTCTAGATTAACGATGAGTCGTCATAA
crtY_R2	TTAACGATGAGTCGTCATAA
16UTR-crtZ_F	CATTATGACGACTCATCGTTAAAGTACATCCGAMMGSAGCATCCTTKATGTTGTGGATTTGGAATGC
crtZ_R	GCAGGTCGACTCTAGATTACTTCCCGGATGC
16UTR-trCrBKT_F	AGACAGGTCGACKCCACCCCGCAAAGGAGSATCGKCRATGGGTCCGGGCATC
trCrBKT_R	AGACAGAAGCTTTTACGCCAGCGCCGC
pTacCDFS_inv_F	TGGAATTCGAGCTCGGTACC
pTacCDFS_inv_R	TTCACACAGGAAACAGACCA
vioAB-F	CACACAGGAAACAGACCAATGAAGCATTCTTCCGATATCTGC
vioAB-R	GGTACCGAGCTCGAATTCCATTATCAGGCCTCTCTAGAAAGCTTTCC
vioAB_mid_F	CATTCGATTCCGAAGCCTGGC
vioAB_mid_R	GCCAGGCTTCGGAATCGAATG
vioC-F	CACACAGGAAACAGACCAATGAAAAGAGCAATCATAGTCGG
vioC-R	GGTACCGAGCTCGAATTCCATTATCAGTTGACCCTCCCTATCTTG
vioD-F	CACACAGGAAACAGACCAATGAAGATTCTGGTCATCGGC
vioD-R	GGTACCGAGCTCGAATTCCATTATCAGCGTTGCAGCGCGTAG
vioE-F	CACACAGGAAACAGACCAATGGAAAACCGGGAACCGCC
vioE-R	GGTACCGAGCTCGAATTCCATTACTAGCGCTTGGCGGCGAAG
vioE_frag_F	CCTGATAATGGAATTCGAGCTGACTGCACGGTGCACCAATG
vioE_frag_R	CAGGTCGACTCTAGAGGATCC
vio_frag_F	GCTAGTAATGGAATTCGAGCTGACTGCACGGTGCACCAATG
pTrcCDFS_IV_R	CATTGGTGCACCGTGCAGTC
pTrcCDFS_IV_F	CGAACTCAGAAGTGAAACGCC
cav2_F	TTTCACACAGGAAACAGACCATGGGGCTTGAGACTGAG
cav3_F	TTTCACACAGGAAACAGACCATGATGGC GAAGAGCAT
BamHIcut_GR	GCAGGTCGACTCTAGAG
cav2_BamHI_F	TTAACTGGATCCTTTCACACAGGAAACAGACCATGGGGCTTGAGACTGAGAAG
cav3_PstI_F	TTAACTCTGCAGTTTCACACAGGAAACAGACCATGATGGCCGAAGAGCATACC
PstI_HindIII_Gib_R	CATCCGCCAAAACAGCCAAGCTTG

plsB_GF	CTAGAGTCGACCTGCAGTTTCACACAGGAAACAGACCATGTCCGGCTGGCCACGA
plsB_GR	TCTGTTTCCTGTGTGAAATTACCCTTCGCCCTGCGTC
plsC_GF	GAAGGGTAATTTCACACAGGAAACAGACCATGCTATATATCTTTCGTCTTATTATTACC
plsC_GR	CCGCCAAAACAGCCAAGCTTTTAAACTTTTCCGGCGGC
sRNAdouble_IV_F	GCACATGTTTGATTTATAAGGG
sRNAdouble_IV_R	CAGCACATTTGAGATCTAGTGG
sRNAdouble_Gib_F	CACTAGATCTCAAATGTGCTGGAATTCTAACACCGTGCGTG
sRNAdouble_Gib_R	CCTTATAAATCAAACATGTGCGGCGAATTGGGTACCTATAAAC
ptrc-cav1_GF	ATGTGACAGCTTATCGCATGCTTGACAATTAATCATCCGG
ptrc-cav1_GR	CCTGGGTTTACCTAGGCATGCTTATATTTCTTTCTGCAAGTTG
ptrc-plsBC_GR	CCTGGGTTTACCTAGGCATGCTTAAACTTTTCCGGCGGCTTC

[a] Underlines denote restriction sites.

Table S3. 5'UTR library sequences and 5'UTR sequences from the selected strains

Gene	5'UTR library sequences [a]
crtE	CGYTCMGCGGAAAGRAGCATCGWCC
crtB	ARCCTTGTTCAAAGGMSYATCTAGG
crtI	GAACCGTTCGAAWGSAGCRTMCAAG
crtY	CCTTCCTCCAWAAGRAGCATCMAST
crtZ	AGTACATCCGAMMGSAGCATCCTTK
trCrBKT	KCCACCCCGCAAAGGAGSATCGKCR

Strain	5'UTR sequences of corresponding genes from the selected strains		
	crtE	*crtB*	*crtI*
LYC2	CGCTCCGCGGAAAGGAGCATCGTCC	AGCCTTGTTCAAAGGACCATCTAGG	GAACCGTTCGAAAGGAGCATCCAAA
LYC33	CGTTCAGCGGAAAGAAGCATCGTCC	AACCTTGTTCAAAGGAGTATCTAGG	GAACCGTTCGAAAG GAGGTACAAG
LYC40	CGCTCAGCGGAAAGGAGCATCGTCC	AGCCTTGTTCAAAGGAGTATCTAGG	GAACCGTTCGAATGGAGCATACAAG
LYC41	CGCTCCGCGGAAAGGAGCATCGTCC	AGCCTTGTTCAAAGGAGCATCTAGG	GAACCGTTCGAATG GAGCGTCCAAG
LYC56	CGTTCCGCGGAAAGAAGCATCGTCC	AGCCTTGTTCAAAGGATATCTAGG	GAACCGTTCGAAAG GAGCATCCAAG
LYC60	CGCTCAGCGGAAAGAAGCATCGACC	AGCCTTGTTCAAAGGAGTATCTAGG	GAACCGTTCGAATG GAGCGTACAAG
LYC62	CGCTCAGCGGAAAGAAGCATCGTCC	AGCCTTGTTCAAAGGACCATCTAGG	GAACCGTTCGAAAG GAGCGTCCAAG
LYC71	CGCTCCGCGGAAAGGAGCATCGTCC	AGCCTTGTTCAAAGGCGTATCTAGG	GAACCGTTCGAATG GAGCGTACAAG
LYC79	CGCTCAGCGGAAAGAAGCATCGACC	AGCCTTGTTCAAAGGACCATCTAGG	GAACCGTTCGAATGGAGCGTCCAAG
LYC80	CGTTCCGCGGAAAGGAGCATCGTCC	AGCCTTGTTCAAAGGAGTATCTAGG	GAACCGTTCGAATGGAGCGTACAAG
	crtY		
BTC1	CCTTCCTCCATAAGAAGCATCAACT		
BTC4	CCTTCCTCCAAAAGAAGCATCCACT		
BTC17	CCTTCCTCCATAAGAAGCATCAACT		
	crtY	*crtZ*	
ZEA17	CCTTCCTCCATAAGGAGCATCAAGT	AGTACATCCGACCGCAGCATCCTTT	
ZEA20	CCTTCCTCCATAAGGAGCATCAACT	AGTACATCCGACAGCAGCATCCTTT	
ZEA32	CCTTCCTCCATAAGGAGCATCAACT	AGTACATCCGAAAGCAGCATCCTTG	
ZEA33	CCTTCCTCCATAAGAAGCATCAACT	AGTACATCCGAACGCAGCATCCTTT	
ZEA37	CCTTCCTCCATAAGGAGCATCAACT	AGTACATCCGAAAGCAGCATCCTTT	
	crtY	*crtZ*	*trCrBKT*
ATX40	CCTTCCTCCATAAGAAGCATCCAGT	AGTACATCCGACCGGAGCATCCTTT	GCCACCCCGCAAAGGAGCATCGTCG
ATX55	CCTTCCTCCATAGAAGCATCCACT	AGTACATCCGACCGGAGCATCCTTG	GCCACCCCGCAAAGGAGGATCGTCA
ATX65	CCTTCCTCCATAAGAGCATCAAGT	AGTACATCCGAAAGCAGCATCCTTT	GCCACCCCGCAAAGGAGGATCGTCA
ATX68	CCTTCCTCCATAAGAAGCATCAAGT	AGTACATCCGAACGGAGCATCCTTG	GCCACCCCGCAAAGGAGGATCGTCA
ATX70	CCTTCCTCCAAAAGAAGCATCCAGT	AGTACATCCGAAAGCAGCATCCTTT	GCCACCCCGCAAAGGAGGATCGTCA
ATX72	CCTTCCTCCATAAGAAGCATCCAGT	AGTACATCCGAACGCAGCATCCTTT	GCCACCCCGCAAAGGAGGATCGGCA
ATX73	CCTTCCTCCATAAGAAGCATCAAGT	AGTACATCCGAACGGAGCATCCTTG	GCCACCCCGCAAAGGAGGATCGTCA
ATX81	CCTTCCTCCATAAGAAGCATCCACT	AGT ACATCCGACA GCAGCATCCTTT	GCCACCCCGCAAAGGAGGATCGTCG
ATX121	CCTTCCTCCATAAGAAGCATCCAGT	AGT ACATCCGACCGGAGCATCCTTT	GCCACCCCGCAAAGGAGCATCGTCG
ATX171	CCTTCCTCCATAAGAAGCATCCAGT	AGTACATCCGAAAGGAGCATCCTTT	TCCACCCCGCAAAGGAGCATCGGCG

[a] Mixed Bases: M, A+C; R, A+G; W, A+T; Y, C+T; S, G+C; and K, G+T

Table S4. List of sRNA knockdown target genes used for morphology engineering or OMV formation

No.	Target gene	Protein function	Essentiality [a)]
**Morphology engineering**			
1	*rodZ*	transmembrane component of cytoskeleton	NE
2	*ftsA*	ATP-binding cell division protein involved in recruitment of FtsK to Z ring	E
3	*ftsB*	cell division protein	E
4	*ftsI*	transpeptidase involved in septal peptidoglycan synthesis	E
5	*ftsL*	membrane bound cell division protein at septum containing leucine zipper motif	E
6	*ftsQ*	membrane anchored protein involved in growth of wall at septum	E
7	*ftsW*	integral membrane protein involved in stabilising FstZ ring during cell division	E
8	*ftsZ*	GTP-binding tubulin-like cell division protein	E
9	*minD*	membrane ATPase of the MinC-MinD-MinE system	E
10	*mrdA*	transpeptidase involved in peptidoglycan synthesis	E
11	*mrdB*	cell wall shape-determining protein	E
12	*mreB*	cell wall structural complex MreBCD, actin-like component MreB	E
13	*mreC*	cell wall structural complex MreBCD transmembrane component MreC	E
14	*zipA*	cell division protein involved in Z ring assembly	E
15	*murE*	UDP-N-acetylmuramoyl-L-alanyl-D-glutamate:meso- diaminopimelate ligase	E
16	*pbpC*	fused transglycosylase and transpeptidase	NE
**Targets for OMV formation**			
1	*rseA*	Inhibitor of σE	NE
2	*rseB*	Inhibitor of σE	E
3	*rffD*	Genes involved in enterobacterial common antigen (ECA) pathway	E
4	*rffC*	Genes involved in ECA pathway	NE
5	*rffA*	Genes involved in ECA pathway	NE
6	*ompR*	Response regulator for *ompC* and *ompF*	NE
7	*gmhB*	Genes involved in lipopolysaccharide (LPS) pathway	NE
8	*lpxL*	Genes involved in LPS pathway	NE
9	*lpxM*	Genes involved in LPS pathway	NE
10	*ompA*	Outer membrane protein A	NE
11	*ompC*	Outer membrane porin	NE
12	*rfaB*	Genes involved in LPS pathway	E
13	*rfaC*	Genes involved in LPS pathway	NE
14	*rfaD*	Genes involved in LPS pathway	NE
15	*rfaE*	Genes involved in LPS pathway	NE
16	*waaG/rfaG*	LPS core biosynthesis; glucosyl transferase	NE
17	*rfaI*	Genes involved in LPS pathway	NE
18	*rfaJ*	Genes involved in LPS pathway	E
19	*rfaK*	Genes involved in LPS pathway	E
20	*rfaP*	Genes involved in LPS pathway	E
21	*rfaQ*	Genes involved in LPS pathway	E
22	*rfaY*	Genes involved in LPS pathway	E
23	*rfbA*	Genes involved in ECA pathway	NE
24	*rffH*	Genes involved in ECA pathway	E
25	*wzxE*	Inner membrane translocase for a component of ECA	NE
26	*pnp*	Polynucleotide phosphorylase	NE

[a)] E, essential; NE, non-essential; essentiality was determined upon the growth of *E. coli* in minimal medium supplemented with simple carbon sources or in LB medium when the corresponding gene was knocked out, as is reported in the metacyc database.

Table S5. Titers, productivities, and contents of rainbow colorants obtained by fed-batch fermentations

Strain [a)]	Carbon source	Titer ($g\ l^{-1}$) [b)]	Productivity ($mg\ l^{-1}\ h^{-1}$)	Content ($mg\ gDCW^{-1}$)
BTC1 (pWAS-anti-*rffDrfaD*)	Glucose	295 $mg\ l^{-1}$	6.42	4.17
BTC1 (pWAS-anti-*rffDrfaD*)	Glucose	291 $mg\ l^{-1}$	6.32	3.99
BTC1 (pWAS-anti-*rffDrfaD*)	Glycerol	343 $mg\ l^{-1}$	4.83	4.83
BTC1 (pWAS-anti-*rffDrfaD*)	Glycerol	298 $mg\ l^{-1}$	4.19	4.19
ZEA20 (pWAS-anti-*rffDrfaD*)	Glycerol	218 $mg\ l^{-1}$	3.33	3.33
ZEA20 (pWAS-anti-*rffDrfaD*)	Glycerol	204 $mg\ l^{-1}$	2.83	2.83
ATX68 (pWAS-anti-*rffDrfaD*)	Glycerol	322 $mg\ l^{-1}$	3.93	3.93
ATX68 (pWAS-anti-*rffDrfaD*)	Glycerol	283 $mg\ l^{-1}$	3.29	3.29
DVIO (pWAS-anti-*rfaI-cav1*)	Glycerol	6.81 (+0.33)	92.2	595
DVIO (pWAS-anti-*rfaI-cav1*)	Glycerol	6.25 (+0.52)	80.6	573
DVIO (pWAS-anti-*rfaI-cav1*)	Glucose	10.8 (+0.53)	105	535
DVIO (pWAS-anti-*rfaI-cav1*)	Glucose	11.0 (+0.16)	114	362
VIO (pWAS-anti-*rfaI-cav1*)	Glucose	6.46 (+0.23) [c)]	54.1	132
VIO (pWAS-anti-*rfaI-cav1*)	Glucose	4.93 (+0.75) [d)]	54.1	336
PDVIO (pWAS-anti-*rfaI*)	Glucose	0.832 (+0.023)	10.3	45.7
PDVIO (pWAS-anti-*rfaI*)	Glucose	0.833 (+0)	12.2	30.3
PVIO (pWAS-anti-*rfaI-cav1*)	Glucose	1.23 (+0.07)	13.9	26.2
PVIO (pWAS-anti-*rfaI-cav1*)	Glucose	1.30 (+0.24)	18.3	28.7

[a)] Each reproduced result is provided below each main fermentation result.
[b)] The titers for violacein derivatives are directly sampled from the fermenter and thus do not contain the aggregates described in Text S1; titers in parentheses are measured by dissolving the aggregates inside the fermenters (Text S1).
[c)] 1.39 $g\ l^{-1}$ of deoxyviolacein was also produced.
[d)] 0.80 $g\ l^{-1}$ of deoxyviolacein was also produced.

Movie S1 (separate file). A time-lapse movie of the whole fermentation process of BTC1 (pWAS-anti-*rfaDrffD*). The video represents the fermentation process for β-carotene production shown in Figure 4B which is compressed into 30 seconds using HitFilm Express.

Movie S2 (separate file). A time-lapse movie showing the whole fermentation process of VIO (pWAS-anti-*rfaI-cav1*). The video represents the fermentation process for violacein production shown in Figure 4F which is compressed into 30 seconds. As the camera automatically changed the exposure settings as time proceeded (the portion of darker pixels increased due to the increased volume of the culture), brightness was manually adjusted using HitFilm Express to display the overall color of the culture as consistent as possible.

SI References

[1] S. W. Seo, J. S. Yang, H. S. Cho, J. Yang, S. C. Kim, J. M. Park, S. Kim, G. Y. Jung, *Sci. Rep.* **2014**, *4*, 4515.

[2] H. S. Choi, S. Y. Lee, T. Y. Kim, H. M. Woo, *Appl. Environ. Microbiol.* **2010**, *76*, 3097.

[3] J. Du, D. Yang, Z. W. Luo, S. Y. Lee, *J. Biotechnol.* **2018**, *267*, 19.

[4] D. Na, S. M. Yoo, H. Chung, H. Park, J. H. Park, S. Y. Lee, *Nat. Biotechnol.* **2013**, *31*, 170.

[5] S. Y. Lee, J. W. Lee, H. Song, J. M. Kim, S. Choi, J. H. Park, **2008**, US patant 20110269183.

[6] D. Yang, W. J. Kim, S. M. Yoo, J. H. Choi, S. H. Ha, M. H. Lee, S. Y. Lee, *Proc. Natl. Acad. Sci. U.S.A.* **2018**, *115*, 9835.

[7] R. Caspi, R. Billington, I. M. Keseler, A. Kothari, M. Krummenacker, P. E. Midford, W. K. Ong, S. Paley, P. Subhraveti, P. D. Karp, *Nucleic Acids Res.* **2020**, *48*, D445.
